# Chiral ammonium betaine-catalyzed asymmetric Mannich-type reaction of oxindoles

**DOI:** 10.3762/bjoc.12.199

**Published:** 2016-09-28

**Authors:** Masahiro Torii, Kohsuke Kato, Daisuke Uraguchi, Takashi Ooi

**Affiliations:** 1 Institute of Transformative Bio-Molecules (WPI-ITbM) and Department of Applied Chemistry, Graduate School of Engineering, Nagoya University, Nagoya 464-8602, Japan; 2 CREST, Japan Science and Technology Agency (JST), Nagoya University, Nagoya 464-8602, Japan

**Keywords:** ammonium betaine, asymmetric catalysis, Mannich reaction, organocatalysis, oxindole

## Abstract

A highly diastereo- and enantioselective Mannich-type reaction of 3-aryloxindoles with *N*-Boc aldimines was achieved under the catalysis of axially chiral ammonium betaines. This catalytic method provides a new tool for the construction of consecutive quaternary and tertiary stereogenic carbon centers on biologically intriguing molecular frameworks with high fidelity.

## Introduction

Chiral indole alkaloids possessing C-3 quaternary indoline frameworks are an important class of biologically relevant molecules, and numerous efforts have been made for the development of reliable synthetic methodologies to enable the installation of the C-3 stereogenic center [1–4]. Among them, the direct stereoselective functionalization of 3-monosubstituted oxindoles is a straightforward method for accessing a wide array of chiral indoline skeletons [5–8]. The most common strategy in this approach is to utilize an oxindole enolate as a nucleophile, because facile deprotonation from the C-3 carbon is ensured by the inductive effect of the α-carbonyl group and by the enolate stability arising from the aromatic character. Accordingly, a number of catalytic methods are available for the asymmetric functionalization of oxindole enolates with various different electrophiles. However, successful examples of Mannich-type reactions with imines are surprisingly limited despite allowing efficient construction of vicinal quaternary and tertiary stereocenters [9–22]. In particular, the application of 3-aryl substituted oxindoles seems problematic; hence, the full potential of this useful carbon–carbon bond formation is yet to be realized [12,14].

**Figure 1:**
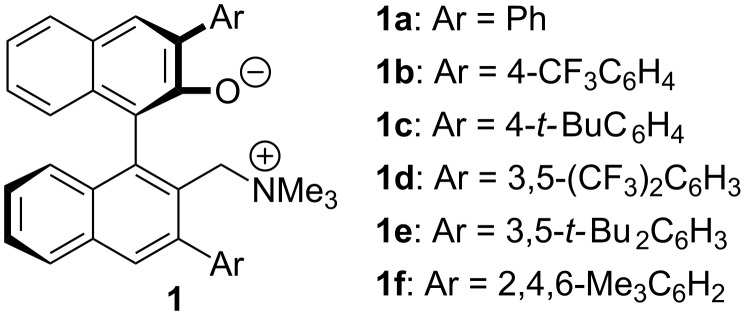
Chiral ammonium betaines.

Ammonium betaines are defined as intramolecular ion-pairing quaternary ammonium salts. In 2008, we employed this structurally distinct molecular scaffold for designing a novel bifunctional organic base catalyst [23], namely axially chiral ammonium betaines of type **1** (Figure 1) [24–25], and uncovered their extraordinary catalytic performance [26–35]. The salient feature of **1** is that, upon abstracting a proton from a pro-nucleophile, the resulting conjugate acid, **1**·H, has the ability to recognize the nucleophilic anion through cooperative electrostatic (ionic) and hydrogen-bonding interactions, thereby precisely controlling the stereochemical outcome of the subsequent bond-forming event. Taking advantage of this unique property, we have developed a series of highly stereoselective transformations, and disclose herein the effectiveness of **1** in solving a challenging problem regarding the rigorous control of the relative and absolute stereochemistry in the asymmetric Mannich-type reaction of 3-aryloxindoles.

## Results and Discussion

As an initial attempt, the reaction of *N*-Boc 3-phenyloxindole (**2a**) with benzaldehyde-derived *N*-Boc imine **3a** [36] was conducted in the presence of a catalytic amount of chiral ammonium betaine **1a** (5 mol %) in toluene with 4 Å molecular sieves (MS 4 Å) at −60 °C. Bond formation occurred smoothly and, after 24 h of stirring, the desired Mannich adduct **4aa** was isolated as a mixture of diastereomers in 90% yield. Although the diastereomeric ratio was moderate (dr = 7.3:1), the enantiomeric excess (ee) of the major isomer was determined to be 98% (Table 1, entry 1). The investigation then focused on the effects of the catalyst structure, primarily on diastereocontrol, which revealed the importance of steric bulk at the periphery of aromatic substituents at the 3,3’-positions of both naphthyl units (Ar), rather than their electronic attributes (Table 1, entries 2–6). For instance, while 4-trifluoromethylphenyl-substituted betaine **1b** had no positive impact on the reaction profile (Table 1, entry 2), the use of **1c**, bearing a 4-*tert*-butylphenyl group, delivered a critical improvement in diastereoselectivity, affording **4aa** quantitatively and establishing consecutive quaternary and tertiary stereocenters with almost complete fidelity (Table 1, entry 3). Further examination of the reactions under the influence of **1d**, having 3,5-bis(trifluoromethyl)phenyl groups, and **1e**, bearing 3,5-bis(*tert*-butyl)phenyl groups, showed similar tendencies, but a considerable decrease in reactivity and selectivity was observed when using **1d** (Table 1, entries 4 and 5). On the other hand, however, the introduction of 2,4,6-trimethylphenyl appendages (**1f**), which extended steric hindrance over the catalytically active sites, eroded the catalytic activity and diastereocontrol (Table 1, entry 6). These observations demonstrated the superior capability of **1c** in facilitating this stereoselective Mannich-type transformation, for which the loading was reduced to 1 mol % without sacrificing reaction efficiency (Table 1, entry 7). It is noteworthy that the present system is scalable; the reaction with 1.0 g of **2a** reached completion within 20 h to afford **4aa** with a similar degree of stereoselectivity (Table 1, entry 8), and subsequent recrystallization furnished 0.83 g of essentially stereochemically pure **4aa**.

**Table 1:** Optimization of catalyst structure.^a^

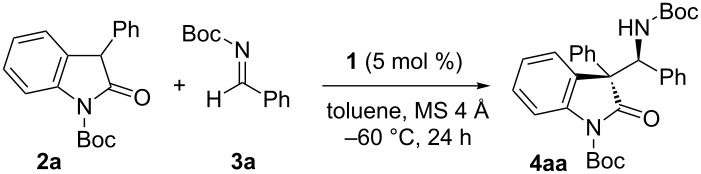				

entry	Ar (**1**)	yield (%)^b^	dr^c^	ee (%)^d^

1	Ph (**1a**)	90	7.3:1	98/28
2	4-CF_3_C_6_H_4_ (**1b**)	>99	7.3:1	98/6
3	4-*t*-BuC_6_H_4_ (**1c**)	>99	>20:1	99/–
4	3,5-(CF_3_)_2_C_6_H_3_ (**1d**)	54	1:1.3	98/−35
5	3,5-*t*-Bu_2_C_6_H_3_ (**1e**)	>99	10:1	98/–
6	2,4,6-Me_3_C_6_H_2_ (**1f**)	73	1.8:1	98/63
7	**1c**^e^	92	>20:1	97/–
8	**1c**^e,f^	>99	>20:1	98/–

^a^Unless otherwise noted, reactions were conducted with 0.1 mmol of **2a**, 0.12 mmol of **3a**, and 5 mol % of **1** in toluene (1.0 mL) containing 100.0 mg of MS 4 Å at −60 °C for 24 h. ^b^Isolated yield was indicated. ^c^The diastereomeric ratio was determined by ^1^H NMR (400 MHz) analysis of crude aliquot. ^d^Enantiomeric excess was analyzed by chiral stationary phase HPLC (DAICEL CHIRALPAK AD-3). Absolute configuration of **4aa** was assigned by analogy to **4ca** (see Figure 2). ^e^1 mol % of **1c** was used. ^f^The reaction was performed on a 1.0 gram scale regarding **2a**.

**Figure 2:**
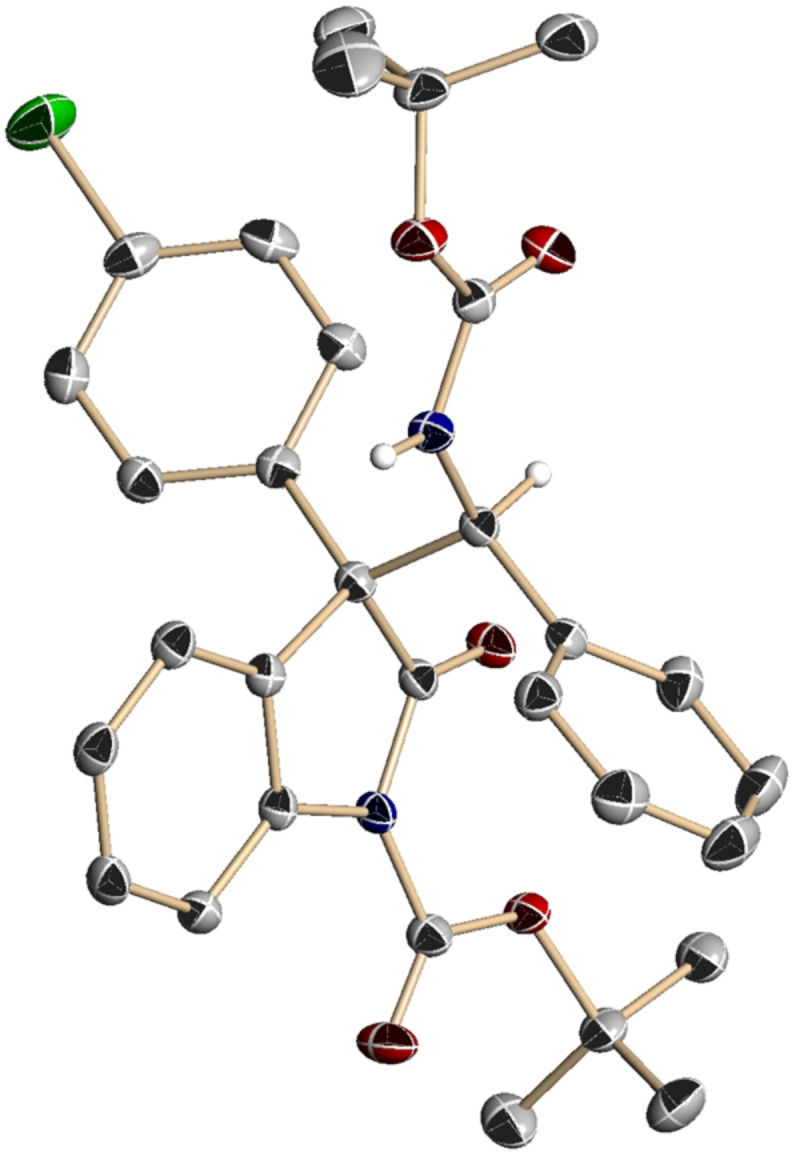
ORTEP diagram of **4ca** (Ellipsoids displayed at 50% probability. Calculated hydrogen atoms except it attaches to stereogenic carbon are omitted for clarity. Black: carbon, Red: oxygen, Blue: nitrogen, Green: chlorine).

Having identified **1c** as an optimal catalyst, the substrate scope of this asymmetric Mannich protocol was explored. As seen in representative results summarized in Table 2, excellent enantioselectivities were generally attained irrespective of the steric and electronic properties of both oxindoles **2** and *N*-Boc aldimines **3**, but reactivity and diastereoselectivity sometimes fluctuated depending on the structure of these substrates. While significant variation in the imine substituents was feasible, the introduction of electron-withdrawing groups at the *meta*-position slightly reduced diastereoselectivity (Table 2, entries 1–4). Sterically demanding 2-tolualdehyde-derived imine **3f** served as a good electrophile and the corresponding Mannich adduct **4af** was isolated as virtually a single stereoisomer (Table 2, entry 5). 3-Thiophenyl aldimine **3g** was also well tolerated, but a substantial decrease in diastereoselectivity was observed in the reaction with 2-furyl aldimine **3h**, owing to the requisite higher reaction temperature (Table 2, entries 6 and 7). Catalysis with **1c** was also applicable to aliphatic imines, which required prolonged reactions and slightly higher catalyst loadings to achieve adequate conversions; the desired adducts, **4ai** and **4aj**, were obtained with high enantioselectivities and moderate diastereoselectivities (Table 2, entries 8 and 9). With respect to oxindoles **2**, the electronic nature of the 3-aryl moiety affected the diastereoselection; the incorporation of electron-deficient aromatics proved beneficial and the presence of electron-rich aryl components seemed detrimental (Table 2, entries 10–14). However, the diastereoselectivity was robust with regard to electronic differences in the oxindole core, and both 5-fluoro- and methoxy-substituted **2g** and **2h** were efficiently converted into **4ga** and **4ha** with rigorous relative and absolute stereocontrol (Table 2, entries 15 and 16). The absolute configuration of **4ca** was unequivocally determined by X-ray crystallographic analysis (Figure 2), and the stereochemistry of the remaining examples was assumed to be analogous.

**Table 2:** Substrate scope.^a^

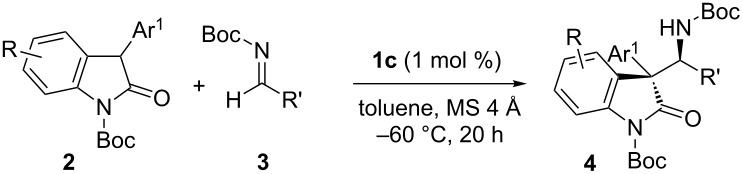						

entry	Ar^1^, R (**2**)	R’ (**3**)	yield (%)^b^	dr^c^	ee (%)^d^	**prod.**

1	Ph, H (**2a**)	4-MeOC_6_H_4_ (**3b**)	96	>20:1	99	**4ab**
2	Ph, H (**2a**)	4-ClC_6_H_4_ (**3c**)	96	>20:1	99	**4ac**
3	Ph, H (**2a**)	3-MeOC_6_H_4_ (**3d**)	92	>20:1	97	**4ad**
4	Ph, H (**2a**)	3-BrC_6_H_4_ (**3e**)	>99	14:1	99	**4ae**
5	Ph, H (**2a**)	2-MeC_6_H_4_ (**3f**)	95	>20:1	99	**4af**
6	Ph, H (**2a**)	3-thiophenyl (**3g**)	90	>20:1	99	**4ag**
7^e^	Ph, H (**2a**)	2-furyl (**3h**)	86	9:1	97	**4ah**
8^f^	Ph, H (**2a**)	Ph(CH_2_)_2_ (**3i**)	55	5:1	98/75	**4ai**
9^g^	Ph, H (**2a**)	Me(CH_2_)_7_ (**3j**)	44	3.5:1	93/60	**4aj**
10	4-MeOC_6_H_4_, H (**2b**)	Ph (**3a**)	96	12:1	99	**4ba**
11	4-ClC_6_H_4_, H (**2c**)	Ph (**3a**)	92	>20:1	97	**4ca**
12	3-MeOC_6_H_4_, H (**2d**)	Ph (**3a**)	89	4:1	98/81	**4da**
13	3-MeC_6_H_4_ (**2e**)	Ph (**3a**)	87	13:1	99	**4ea**
14	3-CF_3_C_6_H_4_, H (**2f**)	Ph (**3a**)	80	>20:1	99	**4fa**
15	Ph, 5-F (**2g**)	Ph (**3a**)	85	>20:1	97	**4ga**
16	Ph, 5-MeO (**2h**)	Ph (**3a**)	89	>20:1	96	**4ha**

^a^Unless otherwise noted, reactions were performed on 0.2 mmol scale with 1.2 equiv of **3a** in the presence of **1c** (1 mol %) and MS 4 Å (100.0 mg) in toluene (1.0 mL) at −60 °C for 24 h. ^b^Isolated yield was reported. ^c^The diastereomeric ratio was determined by ^1^H NMR (400 MHz) analysis of crude aliquot. ^d^Enantiomeric excess of the major isomer was indicated except for entries 8, 9, and 12, which was analyzed by chiral stationary phase HPLC. Absolute configuration of **4ca** was determined by single crystal X-ray diffraction analysis (Figure 2) and that of other **4** was assumed to be analogous. ^e^The reaction was conducted at –40 °C for 110 h. ^f^The reaction was stirred for 117 h. ^g^The reaction time was 72 h.

## Conclusion

In summary, we have clearly demonstrated that chiral ammonium betaine **1c** acts as a uniquely effective catalyst in promoting a Mannich-type reaction between 3-aryloxindoles and *N*-Boc aldimines with high levels of diastereo- and enantioselectivity under mild conditions. This study greatly expands the scope of this mode of stereoselective Mannich-type reaction, which involve the generation of vicinal quaternary and tertiary stereocenters. Further investigations into the potential utility of ammonium betaine catalysis are underway in our laboratory.

## Supporting Information

File 1 Experimental procedures, characterization data, copies of NMR charts and HPLC traces, and X-ray data.

File 2 Crystallographic information file of compound **4ca**.
