# Supplementary material for: Chiral ammonium betaine-catalyzed asymmetric Mannich-type reaction of oxindoles
Source: Beilstein J Org Chem. 2016 Sep 28;12:2099–103. doi: 10.3762/bjoc.12.199 (PMC5082719; doi:10.3762/bjoc.12.199)
Supplement: File 1 — Experimental procedures, characterization data, copies of NMR charts and HPLC traces, and X-ray data. [file Beilstein_J_Org_Chem-12-2099-s001.pdf]

Supporting Information

for

Chiral ammonium betaine-catalyzed asymmetric Mannich-type reaction of oxindoles

Masahiro Torii[1], Kohsuke Kato[1], Daisuke Uraguchi[1] and Takashi Ooi*[1,2]

Address: [1]Institute of Transformative Bio-Molecules (WPI-ITbM) and Department of Applied Chemistry, Graduate School of Engineering, Nagoya University, Nagoya 464-8602, Japan and [2]CREST, Japan Science and Technology Agency (JST), Nagoya University, Nagoya 464-8602, Japan.

Email: Takashi Ooi* - tooi@apchem.nagoya-u.ac.jp

*Corresponding author

Experimental procedures, characterization data, copies of NMR charts and HPLC traces, and X-ray data

General Information: Infrared spectra were recorded on a SHIMADZU IRAffinity-1 spectrometer. ^{1}H NMR spectra were recorded on a JEOL JNM-ECS400 (400 MHz) spectrometer or a JEOL JNM-ECA600 (600 MHz) spectrometer. Chemical shifts are reported in ppm from the solvent resonance ($(CD_3)_2SO$: 2.50 ppm, CD_3CN: 1.94 ppm) or tetramethylsilane (0.00 ppm; $CDCl_3$, CD_3OD) resonance as the internal standard. Data are reported as follows: chemical shift, integration, multiplicity (s = singlet, d = doublet, t = triplet, m = multiplet, br = broad, brd = broad-doublet), and coupling constants (Hz). ^{13}C NMR spectra were recorded on a JEOL JNM-ECS400 (100 MHz) spectrometer or a JEOL JNM-ECA600 (151 MHz) spectrometer with complete proton decoupling. Chemical shifts are reported in ppm from the solvent resonance ($CDCl_3$: 77.16 ppm, CD_3OD: 49.00 ppm, $(CD_3)_2SO$: 39.52 ppm, CD_3CN: 1.32 ppm). ^{19}F NMR spectra were recorded on a JEOL JNM-ECS400 (373 MHz) spectrometer with complete proton decoupling. Chemical shifts are reported in ppm from benzotrifluoride (–64.0 ppm) resonance as the external standard. The high resolution mass spectra were conducted on Thermo Fisher Scientific Exactive (ESI). Analytical thin layer chromatography (TLC) was performed on Merck precoated TLC

plates (silica gel 60 F_{254}, 0.25 mm). Flash column chromatography was performed on Silica gel 60N (spherical neutral, 40–50 μm; Kanto Chemical Co., Inc.) or Chromatorex NH-DM2035 silica gel (Fuji Silysia Chemical Ltd.). Enantiomeric excesses were determined by HPLC analysis using chiral columns [ϕ 4.6 mm × 250 mm, DAICEL CHIRALPAK AD-3 (AD-3), CHIRALPAK AZ-3 (AZ-3), CHIRALPAK IC-3 (IC-3), CHIRALPAK IE-3 (IE-3), CHIRALPAK IF-3 (IF-3), and CHIRALCEL OZ-3 (OZ-3)] with hexane (H), 2-propanol (IPA), and ethanol (EtOH) as eluent.

Toluene was supplied from Kanto Chemical Co., Inc. as "Dehydrated" and further purified by passing through neutral alumina under nitrogen atmosphere. Betaines[1], oxindoles[2], and *N*-Boc imines[3] were prepared by following literature procedure. Powdered 4 Å molecular sieves (MS 4 Å) was supplied by Sigma-Aldrich Co. Other simple chemicals were purchased and used as such.

---

1 (a) Uraguchi, D.; Koshimoto, K.; Ooi, T. *Chem. Commun.* **2010**, *46*, 300. (b) Oyaizu, K.; Uraguchi, D.; Ooi, T. *Chem. Commun.* **2015**, *51*, 4437.

2 (a) Hamashima, Y.; Suzuki, T.; Takano, H.; Shimura, Y.; Sodeoka, M. *J. Am. Chem. Soc.* **2005**, *127*, 10164. (b) Duan, S.-W.; An, J.; Chen, J.-R.; Xiao, W.-J. *Org. Lett.* **2011**, *13*, 2290.

3 (a) Wenzel, A. G.; Jacobsen, E. N. *J. Am. Chem. Soc.* **2002**, *124*, 12964. (b) Song, J.; Wang, Y.; Deng, L. *J. Am. Chem. Soc.* **2006**, *128*, 6048.

Experimental section:

Characterization of betaine precursor 1c·HCl and betaine 1c:

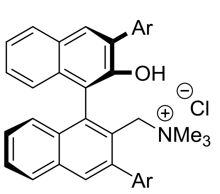

(Ar = 4-*t*-BuC$_6$H$_4$)

1c·HCl: ^{1}H NMR (600 MHz, $(CD_3)_2SO$, 80 °C) δ 8.73 (1H, s), 8.12 (2H, t, J = 3.9 Hz), 8.08 (1H, s), 8.02 (1H, d, J = 7.8 Hz), 7.66-7.63 (5H, m), 7.58 (2H, d, J = 7.8 Hz), 7.53 (2H, d, J = 7.8 Hz), 7.43 (1H, t, J = 7.8 Hz), 7.40 (1H, t, J = 7.8 Hz), 7.34 (1H, t, J = 7.8 Hz), 7.27 (1H, d, J = 7.8 Hz), 6.91 (1H, d, J = 7.8 Hz), 4.90 (1H, br), 4.85 (1H, br), 2.52 (9H, s), 1.40 (9H, s), 1.38 (9H, s); ^{13}C NMR (151 MHz, $(CD_3)_2SO$, 80 °C) δ 150.4, 149.7, 140.8, 137.9, 134.7, 133.8, 132.3, 131.5, 130.9, 130.8, 129.3, 128.8, 128.3, 128.2, 127.9, 127.6, 126.9, 126.8, 126.5, 125.4, 124.6, 123.4, 117.7, 64.4, 53.6, 34.0, 33.9, 30.7_7, 30.7_3, five carbon atoms were not found probably due to broadening or overlapping; IR (film) 3439, 2961, 1717, 1622, 1474, 1364, 1267, 1148, 1067, 1026, 839, 752 cm^{-1}; HRMS (ESI) Calcd for $C_{44}H_{48}NO^+$ ($[M–Cl]^+$) 606.3730, Found 606.3718.

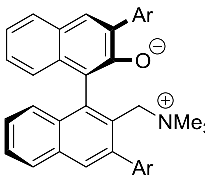

(Ar = 4-*t*-BuC$_6$H$_4$)

1c: ^{1}H NMR (600 MHz, CD_3OD) δ 7.97 (2H, br), 7.89 (1H, br), 7.83 (1H, br), 7,73 (1H, br), 7.62 (3H, br), 7.56 (3H, br), 7.47 (3H, d, J = 7.8 Hz), 7.38-7.20 (2H, br), 7.04 (1H, s), 6.79-6.69 (1H, br), 5.12 (1H, dd, J = 13.2, 29.4 Hz), 4.34 (1H, d, J = 13.2 Hz), 2.61 (5H, s), 2.23 (4H, s), 1.40 (9H, s), 1.36 (9H, s); ^{13}C NMR analysis gave broad spectrum and it was not assignable; IR (film) 3402, 2961, 2361, 1609, 1485, 1423, 1393, 1269, 1152, 1024, 837, 752 cm^{-1}; HRMS (ESI) Calcd for $C_{44}H_{48}NO^+$ ($[M+H]^+$) 606.3730, Found 606.3725.

Representative procedure for asymmetric Mannich-type reaction of oxindole 2a to imine 3a catalyzed by chiral ammonium betaine 1c:

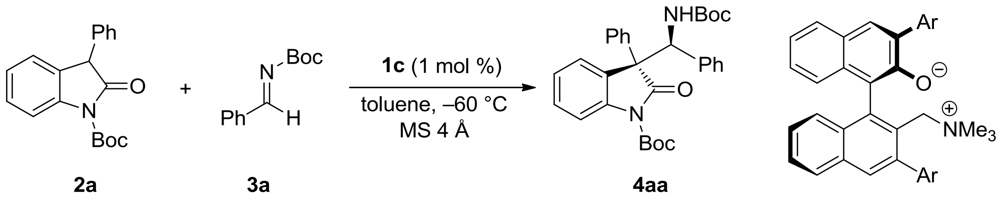

2a **3a** **4aa**

1c (Ar = 4-*t*-BuC$_6$H$_4$)

A magnetic stirrer bar and oven-dried 4 Å molecular sieves (MS 4 Å) (100.0 mg) were placed in an oven-dried test tube under argon (Ar) atmosphere. The test tube was heated with a heat gun under reduced pressure for 3 min and it was refilled with Ar. Then, chiral ammonium betaine **1c** (1.21 mg, 0.0020 mmol) and oxindole **2a** (61.9 mg, 0.2 mmol) were added to the test tube. After cooling to −60 °C, toluene (1.0 mL) and imine **3a** (49.3 mg, 0.24 mmol) were introduced and stirring was continued for 20 h. The reaction was quenched by the addition of a solution of trifluoroacetic acid in toluene (1.0 M, 2.0 μL) and hydrochloric acid (1.0 M, 2.0 mL). The aqueous phase was extracted with ethyl acetate (EA) twice. The combined organic phases were washed with brine, dried over Na_2SO_4, and filtered. All volatiles were removed by evaporation to afford the crude residue, which was analyzed by ^{1}H NMR (600 MHz) to determine the diastereomeric ratio (dr = >20:1). Purification of the residue by column chromatography on Silica gel 60N (H/EA = 10:1 to 5:1 as eluent) gave **4aa** as a mixture of isomers (113.8 mg, 92%). The enantiomeric ratio was determined by chiral stationary phase HPLC (97% ee). **4aa**: HPLC AD-3, H/IPA/EtOH = 48:1:1, flow rate = 0.3 mL/min, λ = 210 nm, 22.8 min (major isomer of major diastereomer), 25.7 min (minor diastereomer), 28.6 min (minor isomer of major diastereomer), 37.7 min (minor diastereomer); ^{1}H

NMR (600 MHz, $CDCl_3$) major diastereomer δ 7.65 (1H, d, J = 7.8 Hz), 7.53 (2H, d, J = 7.8 Hz), 7.48 (1H, brd, J = 7.8 Hz), 7.39-7.28 (5H, m), 7.11 (1H, t, J = 7.8 Hz), 7.04 (2H, t, J = 7.8 Hz), 6.80 (2H, d, J = 7.8 Hz), 5.92 (1H, br), 5.13 (1H, br), 1.47 (9H, s), 1.30 (9H, s); ^{13}C NMR (151 MHz, $CDCl_3$) major diastereomer δ 174.3, 154.9, 148.6, 141.0, 137.1, 136.3, 129.5, 128.8, 128.7, 128.1, 127.9, 127.8, 127.7, 127.4, 125.4, 124.4, 115.5, 84.2, 80.4, 62.5, 59.5, 28.3, 28.1; IR (film) 2978, 1761, 1732, 1695, 1607, 1481, 1368, 1346, 1302, 1287, 1250, 1152, 1059 cm^{-1}; HRMS (ESI) Calcd for $C_{31}H_{34}N_2NaO_5^+$ ($[M+Na]^+$) 537.2360, Found 537.2359.

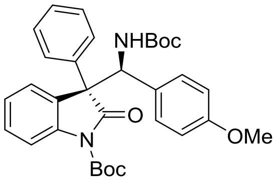

4ab: HPLC IE-3, H/EtOH = 19:1, flow rate = 0.5 mL/min, λ = 210 nm, 25.9 min (minor diastereomer), 30.3 min (minor diastereomer), 44.9 min (minor isomer of major diastereomer), 51.3 min (major isomer of major diastereomer); ^1H NMR (600 MHz, $CDCl_3$) major diastereomer δ 7.68 (1H, d, J = 7.8 Hz), 7.52 (2H, d, J = 7.8 Hz), 7.47 (1H, brd, J = 7.8 Hz), 7.38 (1H, t, J = 7.8 Hz), 7.34 (2H, t, J = 7.8 Hz), 7.32-7.27 (2H, m), 6.71 (2H, d, J = 8.7 Hz), 6.57 (2H, d, J = 8.7 Hz), 5.87 (1H, br), 5.10 (1H, brd, J = 6.0 Hz), 3.68 (3H, s), 1.48 (9H, s), 1.30 (9H, s); ^{13}C NMR (151 MHz, $CDCl_3$) major diastereomer δ 174.4, 159.1, 154.8, 148.6, 141.0, 136.3, 129.4, 129.2, 128.9, 128.7, 128.6, 128.1, 127.5, 125.4, 124.4, 115.6, 113.0, 84.1, 80.2, 62.5, 58.9, 55.2, 28.3, 28.0; IR (film) 2980, 1761, 1732, 1697, 1611, 1514, 1368, 1346, 1304, 1288, 1252, 1153, 1034 cm^{-1}; HRMS (ESI) Calcd for $C_{32}H_{36}N_2NaO_6^+$ ($[M+Na]^+$) 567.2466, Found 567.2454.

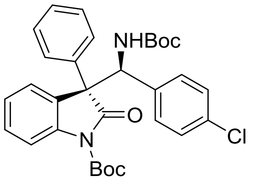

4ac: HPLC IF-3, H/EtOH = 97:3, flow rate = 0.3 mL/min, λ = 210 nm, 23.1 min (minor diastereomer), 26.3 min (minor diastereomer), 28.2 min (minor isomer of major diastereomer), 32.4 min (major isomer of major diastereomer); ^1H NMR (600 MHz, $CDCl_3$) major diastereomer δ 7.67 (1H, d, J = 7.8 Hz), 7.49 (2H, d, J = 7.8 Hz), 7.47 (1H, brd, J = 7.8 Hz), 7.39 (1H, t, J = 7.8 Hz), 7.35 (2H, t, J = 7.8 Hz), 7.32-7.29 (2H, m), 7.02 (2H, d, J = 7.8 Hz), 6.77 (2H, d, J = 7.8 Hz), 5.87 (1H, br), 5.17 (1H, br), 1.49 (9H, s), 1.30 (9H, s); ^{13}C NMR (151 MHz, $CDCl_3$) major diastereomer δ 174.2, 154.8, 148.4, 140.9, 135.9, 135.8, 133.7, 129.6, 129.2, 128.7, 128.6, 128.3, 127.8, 126.9, 125.4, 124.5, 115.6, 84.4, 80.6, 62.2, 59.0, 28.3, 28.0; IR (film) 2980, 1759, 1732, 1695, 1607, 1491, 1368, 1346, 1304, 1287, 1250, 1150, 1098 cm^{-1}; HRMS (ESI) Calcd for $C_{31}H_{33}{}^{35}ClN_2NaO_5^+$ $[(M+Na)^+]$ 571.1970, Found 571.1970.

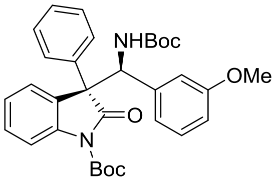

4ad: HPLC OZ-3, H/EtOH = 9:1, flow rate = 0.5 mL/min, λ = 210 nm, 8.3 min (minor diastereomer), 9.4 min (minor diastereomer), 10.2 min (minor isomer of major diastereomer), 12.2 min (major isomer of major diastereomer); ^1H NMR (600 MHz, $CDCl_3$) major diastereomer δ 7.69 (1H, d, J = 8.4 Hz), 7.53 (2H, d, J = 8.4 Hz), 7.47 (1H, brd, J = 8.4 Hz), 7.38 (1H, t, J = 8.4 Hz), 7.35 (2H, t, J = 8.4 Hz), 7.33-7.28 (2H, m), 6.95 (1H, t, J = 8.1 Hz), 6.66 (1H, dd, J = 2.1, 8.1 Hz), 6.40 (1H, d, J = 8.1 Hz), 6.33 (1H, s), 5.90 (1H, br), 5.11 (1H, br), 3.57 (3H, s), 1.48 (9H, s), 1.31 (9H, s); ^{13}C NMR (151 MHz, $CDCl_3$) major diastereomer δ 174.2, 158.9, 154.8, 148.6, 141.1, 138.5, 136.2, 129.4, 128.7, 128.6, 128.1, 128.0, 127.3, 125.4, 124.3, 120.2, 115.6, 114.4, 112.5, 84.2, 80.3, 62.4, 59.5, 55.1, 28.3, 28.0; IR (film) 2978, 1761, 1732, 1695, 1603, 1491, 1368, 1346, 1304, 1288, 1252, 1152, 1053 cm^{-1}; HRMS (ESI) Calcd for $C_{32}H_{36}N_2NaO_6^+$ ($[M+Na]^+$) 567.2466, Found 567.2452.

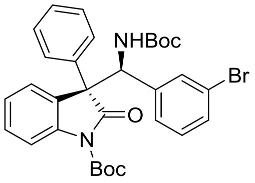

4ae: HPLC OZ-3, H/IPA = 99:1, flow rate = 0.5 mL/min, λ = 210 nm, 12.2 min (minor diastereomer), 16.1 min (minor diastereomer), 22.8 min (minor isomer of major diastereomer), 41.2 min (major isomer of major diastereomer); ^{1}H NMR (600 MHz, $CDCl_3$) major diastereomer *δ* 7.70 (1H, d, *J* = 7.8 Hz), 7.50 (2H, d, *J* = 7.8 Hz), 7.46 (1H, brd, *J* = 7.8 Hz), 7.40 (1H, t, *J* = 7.8 Hz), 7.36 (2H, t, *J* = 7.8 Hz), 7.34-7.30 (2H, m), 7.24 (1H, d, *J* = 7.8 Hz), 6.98 (1H, s), 6.91 (1H, t, *J* = 7.8 Hz), 6.75 (1H, d, *J* = 7.8 Hz), 5.85 (1H, br), 5.14 (1H, br), 1.51 (9H, s), 1.31 (9H, s); ^{13}C NMR (151 MHz, $CDCl_3$) major diastereomer *δ* 174.0, 154.8, 148.5, 140.8, 139.5, 135.8, 131.0, 130.9, 129.7, 129.1, 128.8, 128.6, 128.3, 126.8, 126.4, 125.3, 124.5, 121.7, 115.6, 84.5, 80.6, 62.2, 59.1, 28.3, 28.0; IR (film) 2978, 1761, 1734, 1697, 1607, 1570, 1479, 1368, 1346, 1302, 1288, 1252, 1151, 1059 cm^{-1}; HRMS (ESI) Calcd for $C_{31}H_{33}{}^{79}BrN_2NaO_5^+$ ($[M+Na]^+$) 615.1465, Found 615.1451.

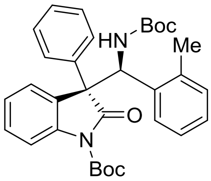

4af: HPLC IE-3, H/IPA = 23:2, flow rate = 0.5 mL/min, λ = 210 nm, 17.6 min (minor diastereomer), 20.7 min (minor isomer of major diastereomer), 22.6 min (major isomer of major diastereomer), 29.2 min (minor diastereomer); ^{1}H NMR (600 MHz, $CDCl_3$) major diastereomer *δ* 7.77 (1H, d, *J* = 7.8 Hz), 7.58 (2H, br), 7.50 (1H, br), 7.45 (1H, t, *J* = 7.8 Hz), 7.37-7.27 (4H, m), 7.07 (1H, d, *J* = 7.8 Hz), 7.02 (1H, t, *J* = 7.8 Hz), 6.72 (1H, t, *J* = 7.8 Hz), 6.35 (1H, br), 5.98 (1H, d, *J* = 7.8 Hz), 5.14 (1H, brd, *J* = 9.6 Hz), 2.57 (3H, s), 1.41 (9H, s), 1.32 (9H, s); ^{13}C NMR (151 MHz, $CDCl_3$) major diastereomer *δ* 173.9, 154.7, 148.6, 141.4, 136.9, 136.7, 136.2, 130.5, 129.6, 128.7, 128.5, 127.9, 127.8, 126.1, 125.7, 125.3, 124.5, 115.5, 83.8, 80.2, 61.8, 53.3, 28.3, 27.9, 20.2, one carbon atom was not found probably due to overlapping; IR (film) 2980, 1763, 1732, 1697, 1607, 1479, 1368, 1348, 1304, 1288, 1252, 1152, 1057 cm^{-1}; HRMS (ESI) Calcd for $C_{32}H_{36}N_2NaO_5^+$ ($[M+Na]^+$) 551.2516, Found 551.2506.

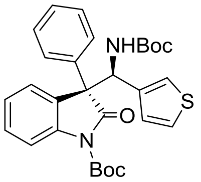

4ag: HPLC IC-3, H/EtOH = 49:1, flow rate = 0.3 mL/min, λ = 210 nm, 23.2 min (minor diastereomer), 25.3 min (minor diastereomer), 32.9 min (minor isomer of major diastereomer), 36.8 min (major isomer of major diastereomer); ^{1}H NMR (600 MHz, $CDCl_3$) major diastereomer *δ* 7.73 (1H, d, *J* = 7.8 Hz), 7.53 (2H, d, *J* = 7.8 Hz), 7.49 (1H, br), 7.39 (1H, t, *J* = 7.8 Hz), 7.34 (2H, t, *J* = 7.8 Hz), 7.29 (2H, t, *J* = 7.8 Hz), 6.96 (1H, dd, *J* = 2.6, 4.8 Hz), 6.81 (1H, d, *J* = 2.6 Hz), 6.36 (1H, d, *J* = 4.8 Hz), 6.06 (1H, br), 5.10 (1H, br), 1.51 (9H, s), 1.31 (9H, s); ^{13}C NMR (151 MHz, $CDCl_3$) major diastereomer *δ* 174.4, 154.8, 148.7, 141.0, 138.3, 136.0, 129.4, 128.6, 128.5, 128.1, 127.6, 126.4, 125.3, 124.8, 124.4, 123.3, 115.5, 84.3, 80.4, 62.2, 55.8, 28.3, 28.1; IR (film) 2978, 2916, 1761, 1732, 1695, 1607, 1481, 1368, 1348, 1302, 1288, 1252, 1152, 1057 cm^{-1}; HRMS (ESI) Calcd for $C_{29}H_{32}N_2NaO_5S^+$ ($[M+Na]^+$) 543.1924, Found 543.1922.

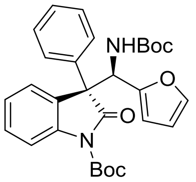

4ah: HPLC AD-3, H/IPA = 19:1, flow rate = 0.5 mL/min, λ = 210 nm, 16.1 min (major isomer of major diastereomer), 21.3 min (minor isomer of major diastereomer), 27.6 min (minor diastereomer), 49.2 min (minor diastereomer); ^{1}H NMR (600 MHz, $CDCl_3$) major diastereomer *δ* 7.80 (1H, d, *J* = 8.4 Hz), 7.51 (2H, brd, *J* = 7.2 Hz), 7.45 (1H, br), 7.37 (1H, dt, *J* = 1.5, 8.0 Hz), 7.31 (2H, t, *J* = 7.2 Hz,), 7.30-7.24 (2H, m), 7.02 (1H, s), 6.10 (1H, dd, *J* =1.8, 3.0 Hz), 6.10 (1H, br), 5.94 (1H, br), 5.16 (1H, brd, *J* = 10.2 Hz), 1.56 (9H, s), 1.32 (9H, s); ^{13}C NMR (151 MHz, $CDCl_3$) major diastereomer *δ* 174.2, 154.7, 150.8, 149.0, 142.0, 140.5, 135.8, 129.2, 128.5, 128.4, 128.0, 127.3, 125.4, 124.3, 115.4, 110.1, 107.6, 84.3, 80.3, 61.6, 54.2, 28.2, 28.1; IR (film) 2980, 1763, 1730, 1697, 1607, 1481, 1368, 1346,

1302, 1287, 1252, 1150, 1061 cm^{-1}; HRMS (ESI) Calcd for $C_{29}H_{32}N_2NaO_6^+$ ($[M+Na]^+$) 527.2153, Found 527.2144.

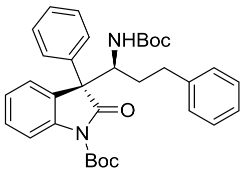

4ai: Purification by column chromatography was performed on Chromatorex NH-DM2035 silica gel (H/EA = 50:1 to 10:1 as eluent). HPLC AD-3, H/IPA = 19:1, flow rate = 0.5 mL/min, λ = 210 nm, 11.3 min (minor diastereomer), 14.1 min (minor isomer of major diastereomer), 16.2 min (major isomer of major diastereomer), 26.6 min (minor diastereomer); ^{1}H NMR (600 MHz, CD_3CN) major diastereomer δ 7.84 (1H, d, J = 7.8 Hz), 7.41-7.38 (2H, m), 7.29-7.23 (8H, m), 7.16 (1H, t, J = 7.8 Hz), 7.11 (2H, d, J = 7.8 Hz), 5.34 (1H, d, J = 10.8 Hz), 4.81 (1H, t, J = 10.8 Hz), 2.66-2.62 (1H, m), 2.54-2.49 (1H, m), 1.57 (11H, m), 1.29 (9H, s); ^{13}C NMR (151 MHz, CD_3CN) major diastereomer δ 176.1, 156.9, 149.8, 142.5, 140.9, 139.1, 129.7, 129.5, 129.3_2, 129.2_8, 128.7, 128.6, 126.9, 126.7, 125.3, 115.8, 85.2, 79.6, 62.6, 56.3, 34.6, 33.1, 28.5, 28.3, one carbon atom was not found probably due to overlapping; IR (film) 2978, 1759, 1732, 1694, 1605, 1497, 1479, 1366, 1348, 1304, 1287, 1248, 1148, 1057, 908 cm^{-1}; HRMS (ESI) Calcd for $C_{33}H_{38}N_2NaO_5^+$($[M+Na]^+$) 565.2673, Found 565.2668.

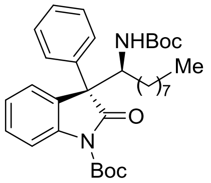

4aj: Purification by column chromatography was performed on Chromatorex NH-DM2035 silica gel (H/EA = 50:1 to 10:1 as eluent). HPLC AZ-3, H/IPA = 97:3, flow rate = 0.5 mL/min, λ = 210 nm, 11.3 min (minor diastereomer), 12.4 min (major isomer of major diastereomer), 14.5 min (minor isomer of major diastereomer), 27.2 min (minor diastereomer); ^{1}H NMR (600 MHz, CD_3CN) major diastereomer δ 7.88 (1H, d, J = 7.8 Hz), 7.42 (2H, t, J = 7.8 Hz), 7.36 (2H, d, J = 7.8 Hz), 7.30-7.25 (4H, m), 5.15 (1H, d, J = 10.8 Hz), 4.78 (1H, t, J = 10.8 Hz), 1.58 (9H, s), 1.26 (13H, m), 1.22 (10H, m), 0.86 (3H, t, J = 7.2 Hz); ^{13}C NMR (151 MHz, CD_3CN) major diastereomer δ 176.2, 157.0, 149.9, 141.0, 139.2, 129.7_4, 129.6_8, 129.3, 128.8, 128.6, 126.7, 125.3, 115.9, 85.2, 79.5, 62.6, 56.6, 32.6, 32.3, 30.1, 29.8, 29.6, 28.5, 28.3, 26.9, 23.3, 14.4; IR (film) 2928, 1759, 1732, 1694, 1607, 1464, 1366, 1348, 1302, 1287, 1248, 1148, 1057, 910 cm^{-1}; HRMS (ESI) Calcd for $C_{33}H_{46}N_2NaO_5^+$ ($[M+Na]^+$) 573.3299, Found 573.3305.

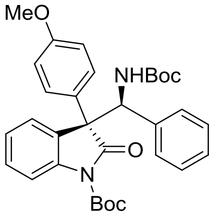

4ba: HPLC OZ-3, H/IPA/EtOH = 48:1:1, flow rate = 0.3 mL/min, λ = 210 nm, 17.7 min (minor diastereomer), 19.1 min (minor diastereomer), 23.7 min (major isomer of major diastereomer), 28.5 min (minor isomer of major diastereomer); ^{1}H NMR (600 MHz, $CDCl_3$) major diastereomer δ 7.64 (1H, d, J = 7.8 Hz), 7.47 (1H, br), 7.44 (2H, d, J = 7.8 Hz), 7.35 (1H, t, J = 7.8 Hz), 7.29 (1H, t, J = 7.8 Hz), 7.09 (1H, t, J = 7.8 Hz), 7.03 (2H, t, J = 7.8 Hz), 6.88 (2H, d, J = 7.8 Hz), 6.80 (2H, d, J = 7.8 Hz), 5.86 (1H, br), 5.19 (1H, d, J = 8.4 Hz), 3.78 (3H, s), 1.47 (9H, s), 1.30 (9H, s); ^{13}C NMR (151 MHz, $CDCl_3$) major diastereomer δ 174.6, 159.4, 154.9, 148.6, 140.8, 137.1, 129.9, 129.3, 128.1, 127.7_1, 127.6_7, 127.6, 125.9, 125.3, 124.3, 115.4, 114.0, 84.0, 80.2, 61.8, 59.4, 55.3, 28.2, 28.0; IR (film) 2978, 1761, 1732, 1697, 1607, 1512, 1368, 1346, 1304, 1288, 1252, 1152, 1034 cm^{-1}; HRMS (ESI) Calcd for $C_{32}H_{36}N_2NaO_6^+$ ($[M+Na]^+$) 567.2466, Found 567.2465.

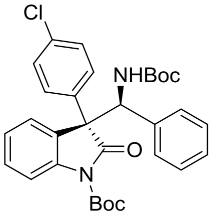

4ca: HPLC AD-3, H/IPA = 97:3, flow rate = 0.5 mL/min, λ = 210 nm, 19.4 min (major isomer of major diastereomer), 24.2 min (minor diastereomer), 26.0 min (minor isomer of major diastereomer), 31.1 min (minor diastereomer); ^{1}H NMR (600 MHz, $CDCl_3$) major diastereomer δ 7.65 (1H, d, J = 8.0 Hz), 7.50 (2H, d, J = 8.0 Hz), 7.47 (1H, br), 7.38 (1H, t, J

= 8.0 Hz), 7.34-7.28 (3H, m), 7.11 (1H, t, J = 8.0 Hz), 7.04 (2H, t, J = 8.0 Hz), 6.78 (2H, d, J = 8.0 Hz), 5.89 (1H, br), 5.22 (1H, br), 1.47 (9H, s), 1.31 (9H, s); ^{13}C NMR (151 MHz, $CDCl_3$) major diastereomer δ 174.0, 154.8, 148.4, 140.9, 136.7, 134.8, 134.2, 130.2, 129.6, 128.6, 128.0, 127.7, 127.6, 126.8, 125.2, 124.5, 115.6, 84.3, 80.5, 62.1, 59.4, 28.2, 28.0; IR (film) 2926, 1763, 1734, 1697, 1607, 1493, 1368, 1346, 1304, 1287, 1252, 1152, 1098 cm^{-1}; HRMS (ESI) Calcd for $C_{31}H_{33}{}^{35}ClN_2NaO_5^+$ ([M+Na]$^+$) 571.1970, Found 571.1961.

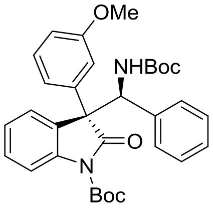

4da: HPLC OZ-3, H/EtOH = 97:3, flow rate = 0.5 mL/min, λ = 210 nm, 10.6 min (minor diastereomer), 12.6 min (minor isomer of major diastereomer), 16.5 min (minor diastereomer), 24.5 min (major isomer of major diastereomer); ^{1}H NMR (600 MHz, $CDCl_3$) major diastereomer δ 7.64 (1H, d, J = 8.4 Hz), 7.46 (1H, br), 7.36 (1H, t, J = 8.1 Hz), 7.29 (1H, t, J = 7.8 Hz), 7.26-7.23 (1H, m), 7.14 (1H, s), 7.10 (1H, t, J = 7.2 Hz), 7.03 (3H, t, J = 7.5 Hz), 6.85 (1H, d, J = 7.8 Hz), 6.80 (2H, d, J = 7.8 Hz), 5.92 (1H, br), 5.17 (1H, br), 3.80 (3H, s), 1.47 (9H, s), 1.31 (9H, s); ^{13}C NMR (151 MHz, $CDCl_3$) major diastereomer δ 174.1, 159.8, 154.9, 148.5, 140.9, 139.9, 138.9, 137.8, 137.0, 129.4, 127.8, 127.7, 127.6, 125.3, 124.4, 121.0, 115.4, 113.9, 113.2, 84.1, 80.3, 62.4, 58.8, 55.4, 28.3, 28.0; IR (film) 2978, 1759, 1732, 1695, 1603, 1493, 1368, 1344, 1302, 1288, 1252, 1150, 1059 cm^{-1}; HRMS (ESI) Calcd for $C_{32}H_{36}N_2NaO_6^+$ ([M+Na]$^+$) 567.2466, Found 567.2456.

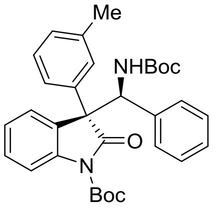

4ea: HPLC AD-3, H/IPA = 97:3, flow rate = 0.5 mL/min, λ = 210 nm, 13.7 min (major isomer of major diastereomer), 15.6 min (minor diastereomer), 18.2 min (minor isomer of major diastereomer), 28.7 min (minor diastereomer); ^{1}H NMR (600 MHz, $CDCl_3$) major diastereomer δ 7.65 (1H, d, J = 7.8 Hz), 7.44 (1H, br), 7.37-7.34 (2H, m), 7.29 (1H, t, J = 7.2 Hz), 7.23-7.20 (2H, m), 7.11 (1H, d, J = 6.6 Hz), 7.09 (1H, d, J = 7.8 Hz), 7.03 (2H, t, J = 7.5 Hz), 6.80 (2H, d, J = 7.2 Hz), 5.92 (1H, br), 5.18 (1H, brd, J = 9.0 Hz), 2.33 (3H, s), 1.47 (9H, s), 1.31 (9H, s); ^{13}C NMR (151 MHz, $CDCl_3$) major diastereomer δ 174.3, 154.9, 148.6, 140.9, 138.2, 137.1, 136.3, 129.3_4, 129.3_0, 128.9, 128.4, 127.7, 127.6, 126.3, 126.2, 125.7, 125.3, 124.4, 115.4, 84.0, 80.2, 62.4, 59.0, 28.2, 28.0, 21.7; IR (film) 2978, 1761, 1732, 1699, 1605, 1481, 1368, 1344, 1302, 1287, 1250, 1150, 1096, 1061, 910 cm^{-1}; HRMS (ESI) Calcd for $C_{32}H_{36}N_2NaO_5^+$ ([M+Na]$^+$) 551.2516, Found 551.2507.

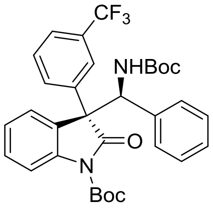

4fa: HPLC IE-3, H/IPA = 19:1, flow rate = 0.5 mL/min, λ = 210 nm, 15.2 min (minor diastereomer), 16.8 min (minor isomer of major diastereomer), 19.1 min (minor diastereomer), 22.7 min (major isomer of major diastereomer); ^{1}H NMR (600 MHz, $CDCl_3$) major diastereomer δ 7.80 (2H, br), 7.68 (1H, d, J = 7.8 Hz), 7.58 (1H, d, J = 7.8 Hz), 7.49 (2H, t, J = 7.8 Hz), 7.41 (1H, dt, J = 1.8, 7.8 Hz), 7.35 (1H, t, J = 7.8 Hz), 7.13 (1H, t, J = 7.8 Hz), 7.05 (2H, t, J = 7.8 Hz), 6.77 (2H, d, J = 7.8 Hz), 5.99 (1H, br), 5.19 (1H, brd, J = 10.2 Hz), 1.47 (9H, s), 1.30 (9H, s); ^{13}C NMR (151 MHz, $CDCl_3$) major diastereomer δ 173.8, 154.7, 148.4, 141.1, 137.5, 136.5, 132.5, 130.8 (q, $J_{F\text{-}C}$ = 32.8 Hz), 129.9, 129.0, 128.1, 127.8, 127.7, 126.6, 125.5, 125.4, 125.0, 124.7, 124.2 (q, $J_{F\text{-}C}$ = 272.8 Hz), 115.7, 84.4, 80.6, 62.5, 59.4, 28.2, 28.0; ^{19}F NMR (373 MHz, $CDCl_3$) δ −62.3; IR (film) 2980, 1761, 1734, 1697, 1607, 1481, 1369, 1346, 1329, 1287, 1252, 1152, 1128, 1080 cm^{-1}; HRMS (ESI) Calcd for $C_{32}H_{33}F_3N_2NaO_5^+$ ([M+Na]$^+$) 605.2234, Found 605.2220.

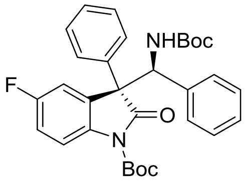

4ga: HPLC IE-3, H/IPA = 19:1, flow rate = 0.5 mL/min, λ = 210 nm, 18.5 min (minor diastereomer), 21.5 min (minor diastereomer), 28.1 min (minor isomer of major diastereomer), 29.8 min (major isomer of major diastereomer); ^{1}H NMR (400 MHz, $CDCl_3$) major diastereomer δ 7.65 (1H, dd, $J_{H\text{-}H}$ = 4.4 Hz, $J_{F\text{-}H}$ = 9.2 Hz), 7.52 (2H, d, J = 8.8 Hz), 7.40-7.28 (3H, m), 7.27 (1H, br), 7.15-7.00 (4H, m), 6.86 (2H, d, J = 7.2 Hz), 5.91 (1H, br), 5.24 (1H, br), 1.47 (9H, s), 1.29 (9H, s); ^{13}C NMR (100 MHz, $CDCl_3$) major diastereomer δ 173.9, 159.7 (d, $J_{F\text{-}C}$ = 245.3 Hz), 154.9, 148.5, 136.9 (d, $J_{F\text{-}C}$ = 6.8 Hz), 135.6, 129.0, 128.7, 128.6, 128.3, 127.9, 127.7_3, 127.6_6, 116.7 (d, $J_{F\text{-}C}$ = 7.7 Hz), 115.9 (d, $J_{F\text{-}C}$ = 22.1 Hz), 113.1 (d, $J_{F\text{-}C}$ = 22.1 Hz), 84.3, 80.5, 62.7, 59.8, 28.2, 28.0, one carbon atom was not found probably due to overlapping; ^{19}F NMR (373 MHz, $CDCl_3$) δ −117.0; IR (film) 2980, 1765, 1732, 1695, 1607, 1483, 1368, 1346, 1298, 1269, 1244, 1150, 1059 cm^{-1}; HRMS (ESI) Calcd for $C_{31}H_{33}FN_2NaO_5^+$ ($[M+Na]^+$) 555.2266, Found 555.2255.

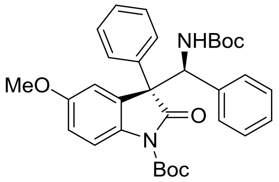

4ha: HPLC OZ-3, H/EtOH = 97:3, flow rate = 0.5 mL/min, λ = 210 nm, 11.2 min (minor diastereomer), 13.5 min (minor diastereomer), 20.2 min (minor isomer of major diastereomer), 28.2 min (major isomer of major diastereomer); ^{1}H NMR (600 MHz, $CDCl_3$) major diastereomer δ 7.59 (1H, d, J = 7.8 Hz), 7.53 (2H, d, J = 7.8 Hz), 7.35 (2H, t, J = 7.8 Hz), 7.30 (1H, t, J = 7.8 Hz), 7.12 (1H, t, J = 7.8 Hz), 7.06 (2H, t, J = 7.8 Hz), 7.03 (1H, s), 6.89 (1H, d, J = 7.8 Hz), 6.84 (2H, d, J = 7.8 Hz), 5.92 (1H, br), 5.17 (1H, brd, J = 7.8 Hz), 3.88 (3H, s), 1.46 (9H, s), 1.30 (9H, s); ^{13}C NMR (151 MHz, $CDCl_3$) major diastereomer δ 174.2, 156.7, 154.8, 148.6, 137.1, 136.2, 134.5, 128.7, 128.6, 128.1, 127.8_3, 127.7_8, 127.6, 125.9, 116.3, 113.2, 112.4, 83.9, 80.3, 62.7, 59.1, 55.9, 28.3, 28.0; IR (film) 2978, 1759, 1728, 1694, 1599, 1487, 1368, 1300, 1279, 1246, 1152, 1036 cm^{-1}; HRMS (ESI) Calcd for $C_{32}H_{36}N_2NaO_6^+$ ($[M+Na]^+$) 567.2466, Found 567.2451.

Crystallographic structure determination: The single crystal, obtained by the procedure described below, was mounted on MicroMesh. Data of X-ray diffraction were collected at 123 K on a Rigaku FR-X with Pilatus 200K with fine-focus sealed tube Mo Kα radiation (λ = 0.71075 Å). An absorption correction was made using Crystal Structure. The structure was solved by direct methods and Fourier syntheses, and refined by full-matrix least squares on F^2 by using SHELXL-2014.[4] All non-hydrogen atoms were refined with anisotropic displacement parameters. A hydrogen atom bonded to a nitrogen atom was located from a difference synthesis and their coordinates and isotropic thermal parameters refined. The other hydrogen atoms were placed in calculated positions and isotropic thermal parameters refined.

Recrystallization of 4ac: Recrystallization of **4ac** was performed by using a $CHCl_3/Et_2O$ solvent system at room temperature.

[4] Sheldrick, G. M. *Acta Cryst.* 2015, **C71**, 3.

Table S1: Crystal data and structure refinement for **4ac**.

Empirical formula	C31 H33 Cl N2 O5	
Formula weight	549.04	
Temperature	123(2) K	
Wavelength	0.71075 Å	
Crystal system	Monoclinic	
Space group	P21	
Unit cell dimensions	a = 8.9878(10) Å	α = 90°
	b = 14.6983(18) Å	β = 90.983(3)°
	c = 10.8669(13) Å	γ = 90°
Volume	1435.4(3) $Å^3$	
Z	2	
Density (calculated)	1.270 Mg/m^3	
Absorption coefficient	0.175 mm^{-1}	
F(000)	580	
Crystal size	0.30 x 0.20 x 0.10 mm^3	
Theta range for data collection	3.2 to 27.5°	
Index ranges	–10<=h<=10, –17<=k<=17, –23<=l<=11	
Reflections collected	9914	
Independent reflections	4860 [R_{int} = 0.0128]	
Completeness to theta = 27.48°	98.4 %	
Absorption correction	Semi-empirical from equivalents	
Max. and min. transmission	0.886 and 1.000	
Refinement method	Full-matrix least-squares on F^2	
Data / restraints / parameters	4860 / 1 / 363	
Goodness-of-fit on F^2	1.080	
Final R indices [I>2sigma(I)]	R_1 = 0.0241, wR_2 = 0.0626	
R indices (all data)	R_1 = 0.0244, wR_2 = 0.0628	
Absolute structure parameter	0.003(11)	
Largest diff. peak and hole	0.237 and –0.295 e.$Å^{-3}$	

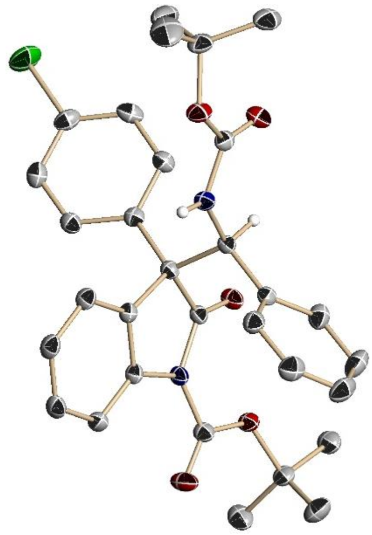

Figure S1: Molecular structure of **4ac**. The thermal ellipsoids of non-hydrogen atoms are shown at the 50% probability level. Blue = nitrogen, green = chlorine, red = oxygen, black = carbon.

Copies of ^1H and ^{13}C NMR spectra:

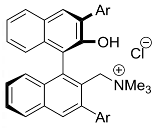

1c·HCl
(Ar = 4-*t*-BuC$_6$H$_4$)

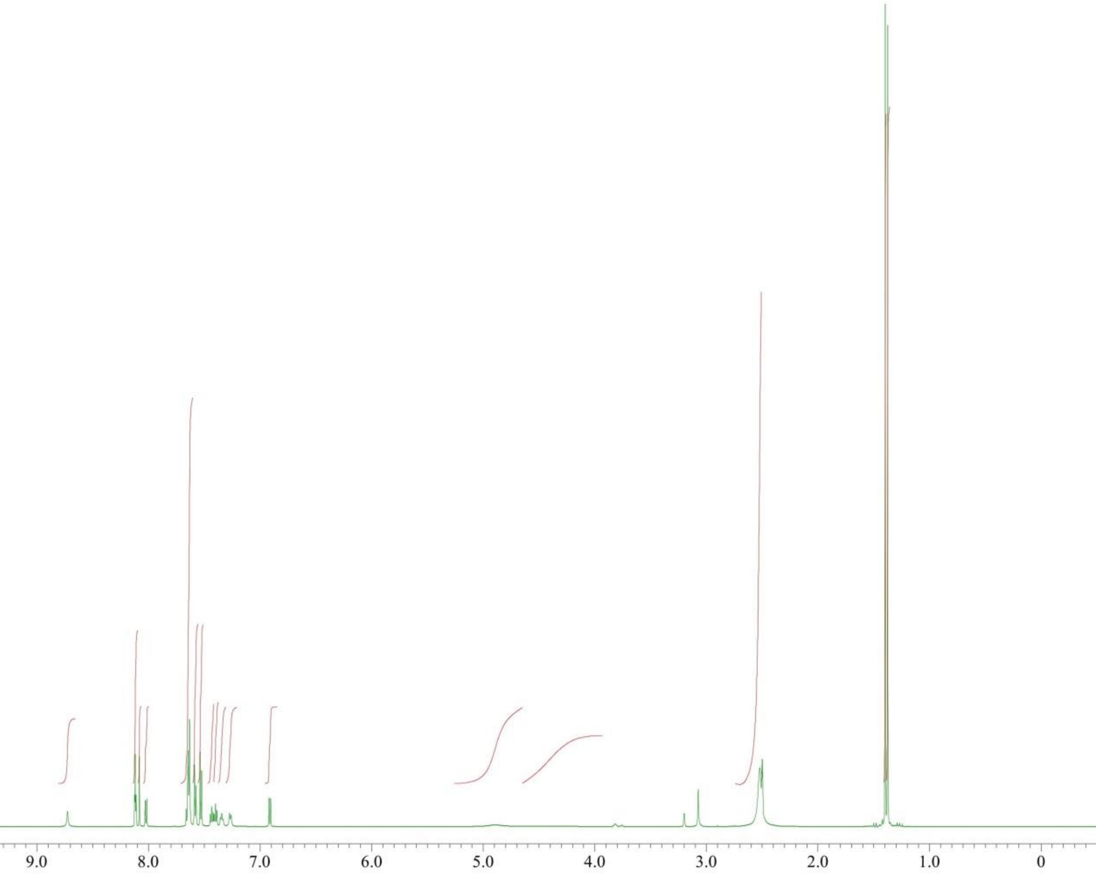

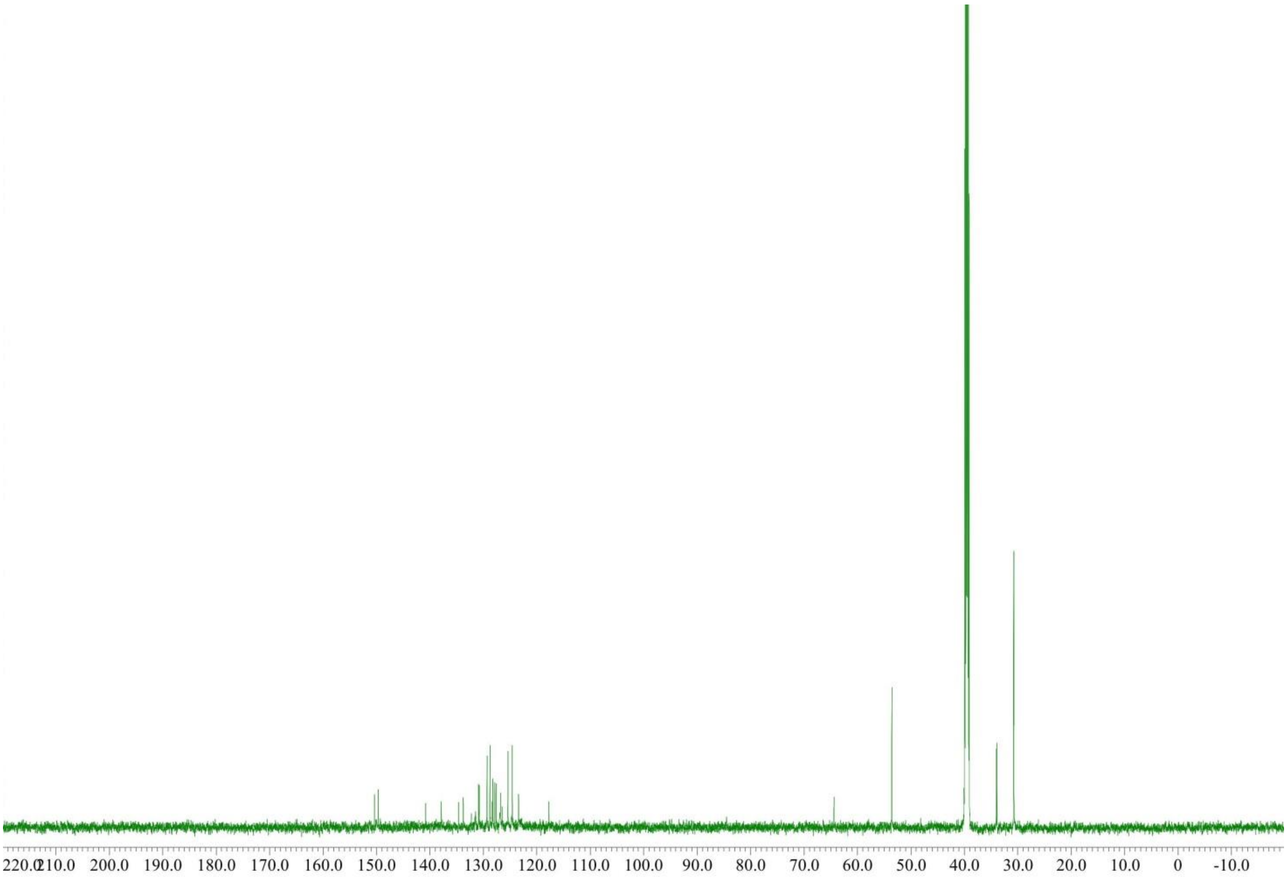

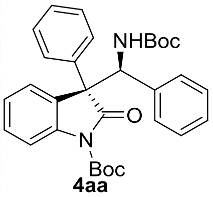
NHBoc
O
N
Boc
4aa

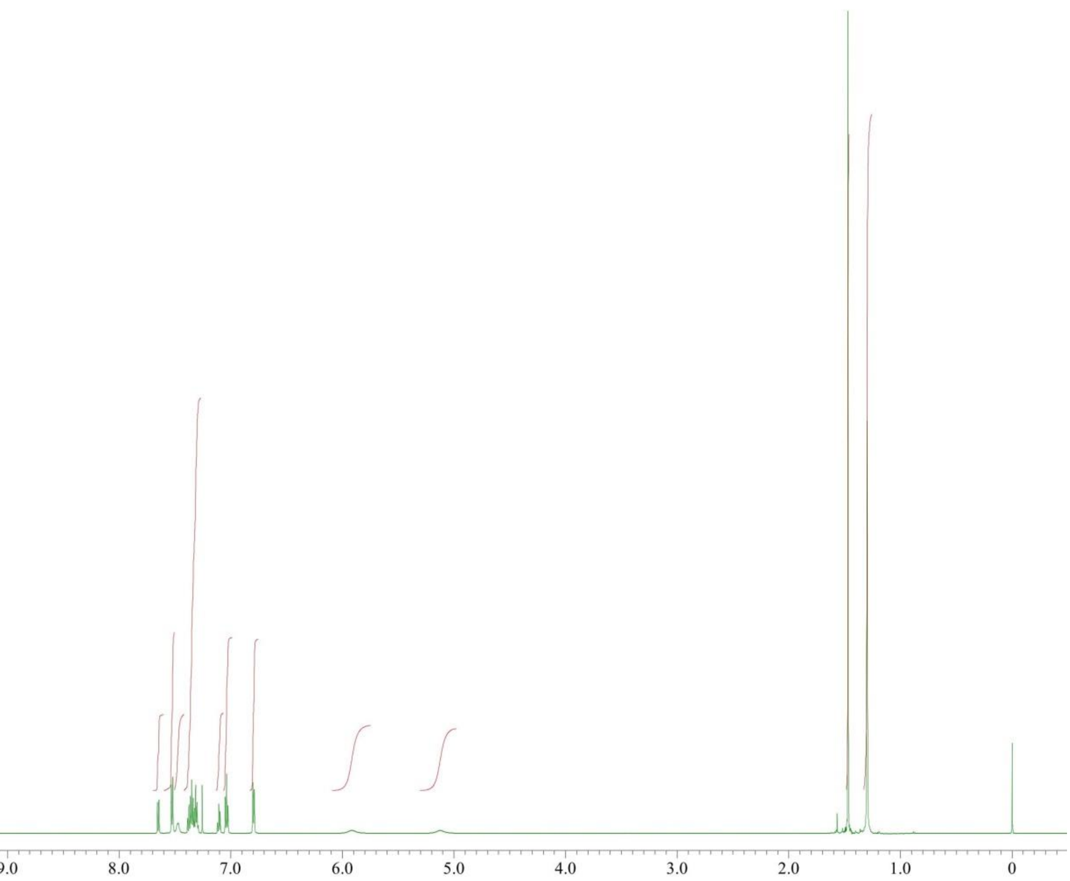
10.0
9.0
8.0
7.0
6.0
5.0
4.0
3.0
2.0
1.0
0

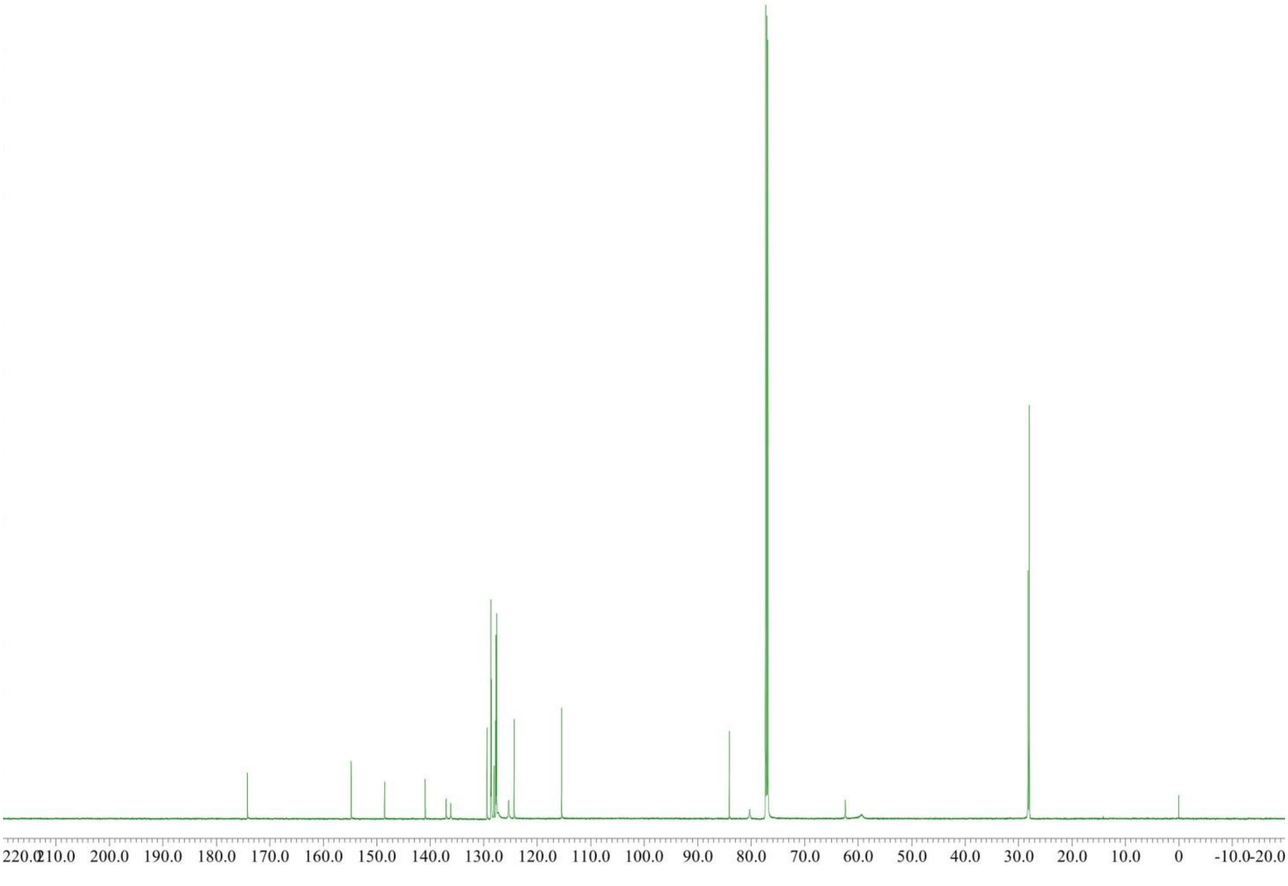
220.0 210.0 200.0 190.0 180.0 170.0 160.0 150.0 140.0 130.0 120.0 110.0 100.0 90.0 80.0 70.0 60.0 50.0 40.0 30.0 20.0 10.0 0 -10.0 -20.0

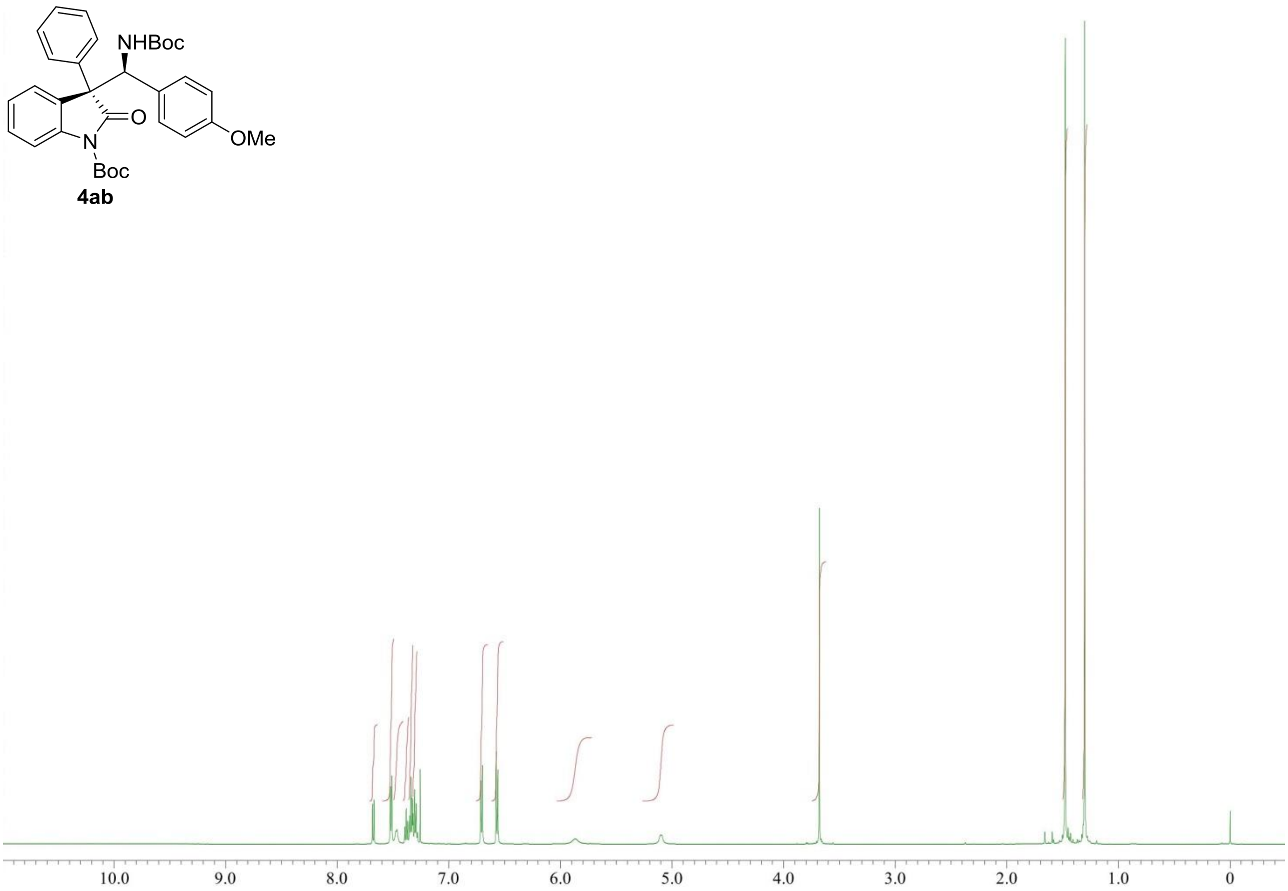
NHBoc
O
N
Boc
OMe
10.0 9.0 8.0 7.0 6.0 5.0 4.0 3.0 2.0 1.0 0

4ab

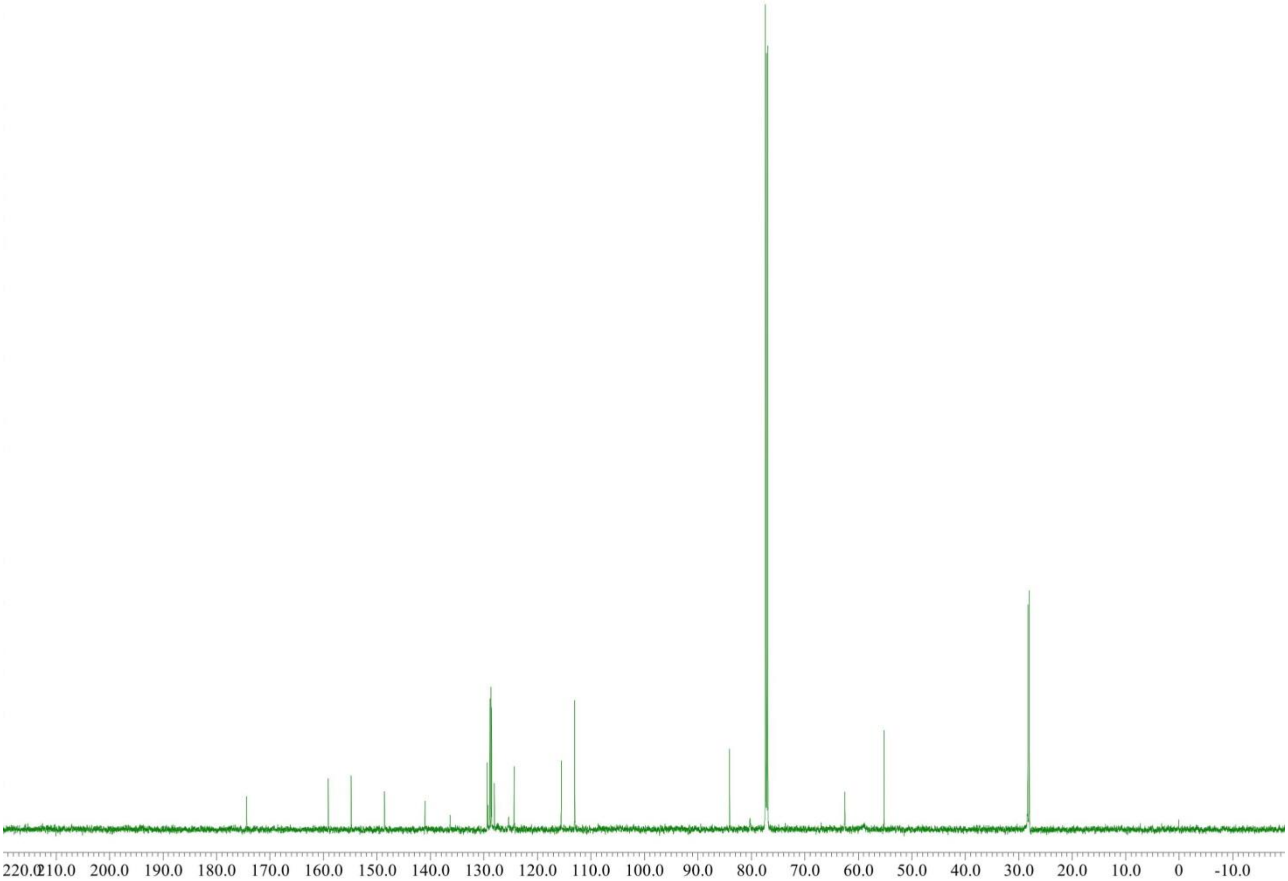
220.0 210.0 200.0 190.0 180.0 170.0 160.0 150.0 140.0 130.0 120.0 110.0 100.0 90.0 80.0 70.0 60.0 50.0 40.0 30.0 20.0 10.0 0 -10.0

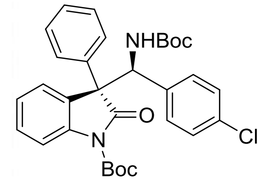

4ac

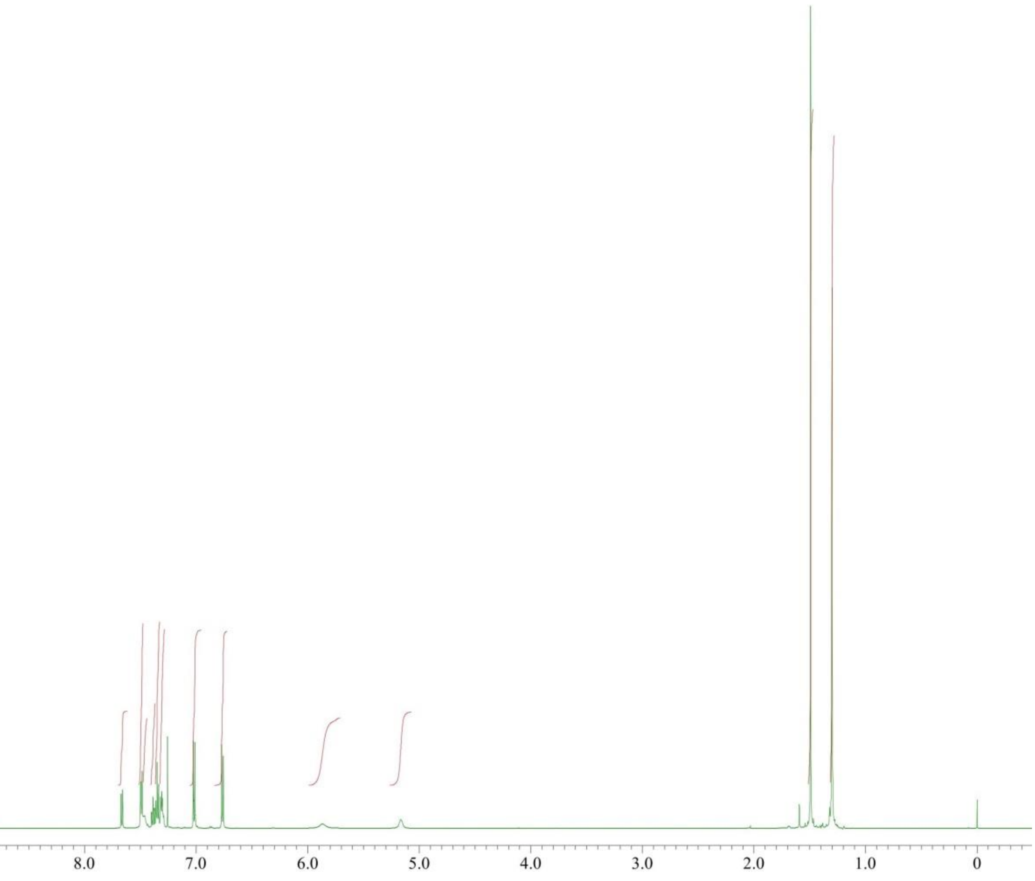

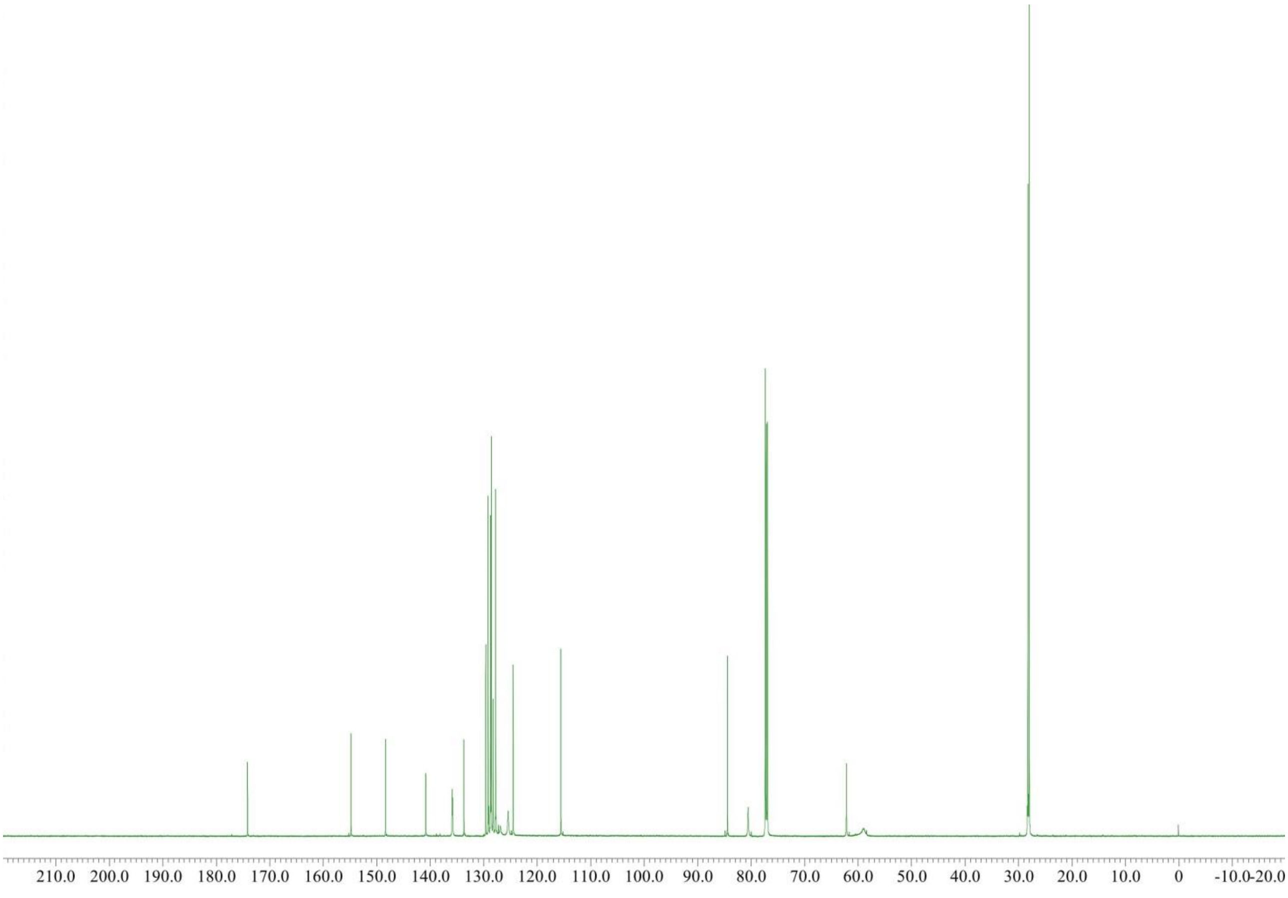

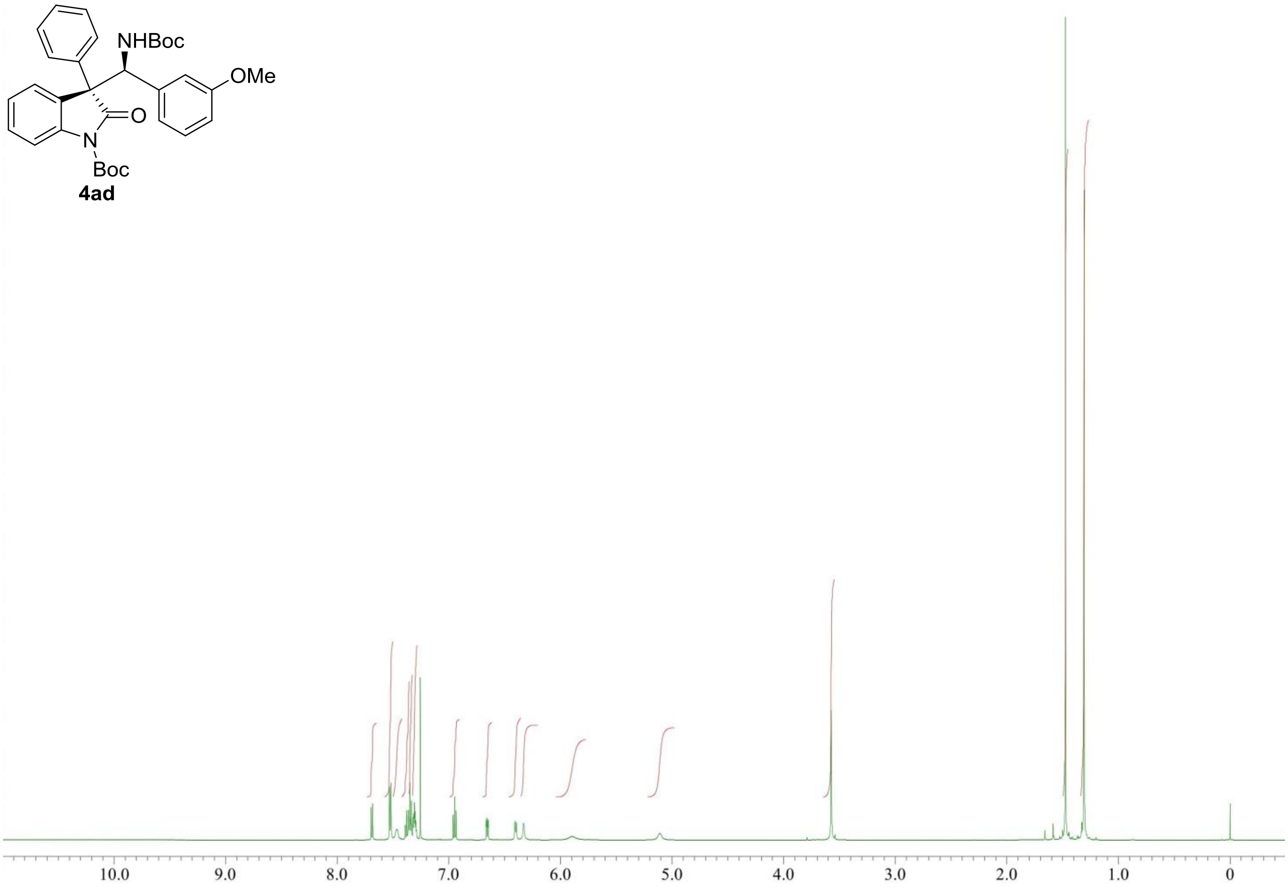
NHBoc
OMe
O
N
Boc
4ad
10.0 9.0 8.0 7.0 6.0 5.0 4.0 3.0 2.0 1.0 0

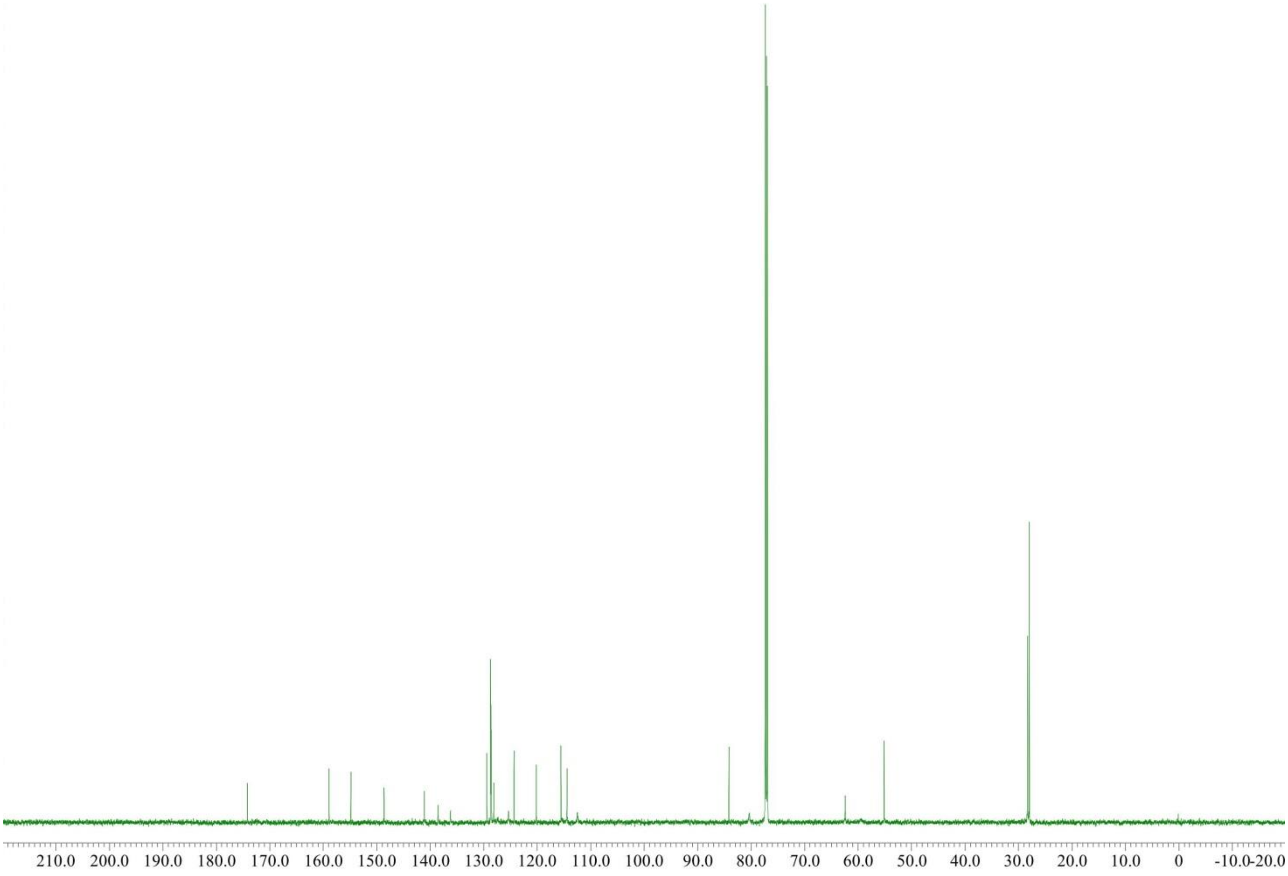
210.0 200.0 190.0 180.0 170.0 160.0 150.0 140.0 130.0 120.0 110.0 100.0 90.0 80.0 70.0 60.0 50.0 40.0 30.0 20.0 10.0 0 -10.0-20.0

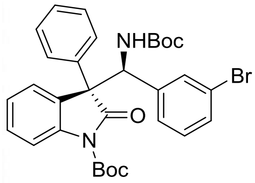
NHBoc
Br
O
N
Boc

4ae

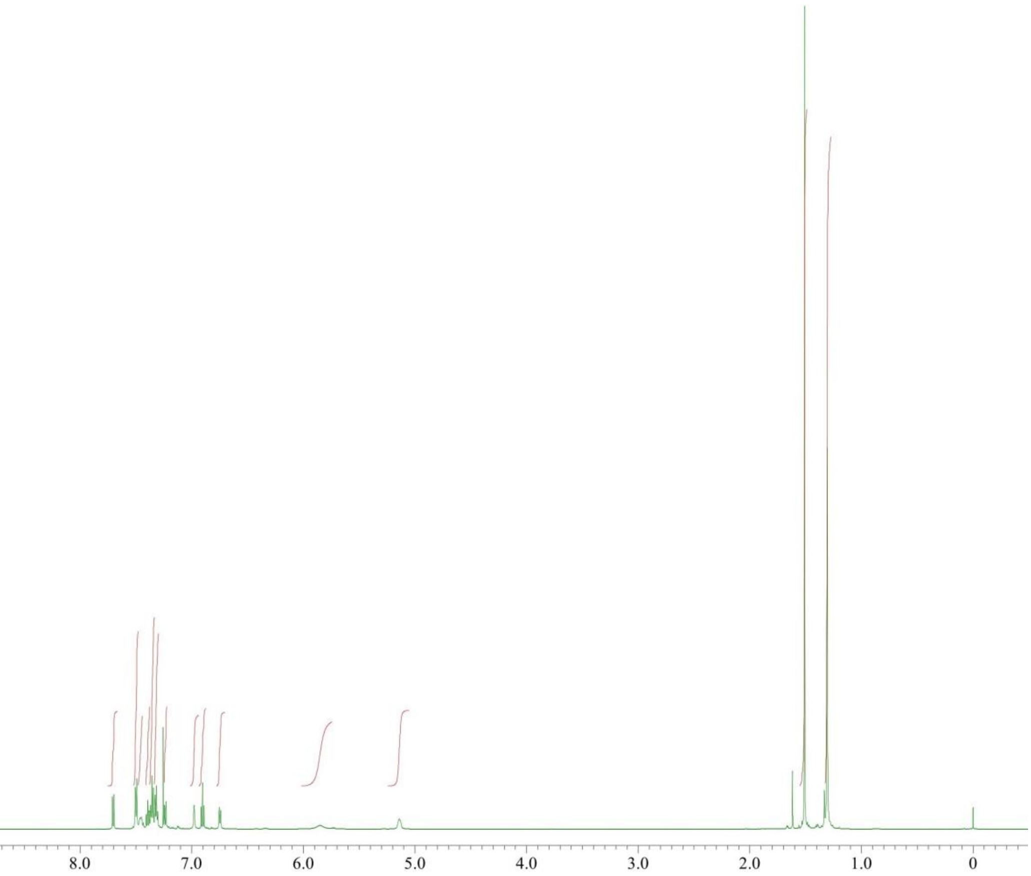
10.0 9.0 8.0 7.0 6.0 5.0 4.0 3.0 2.0 1.0 0

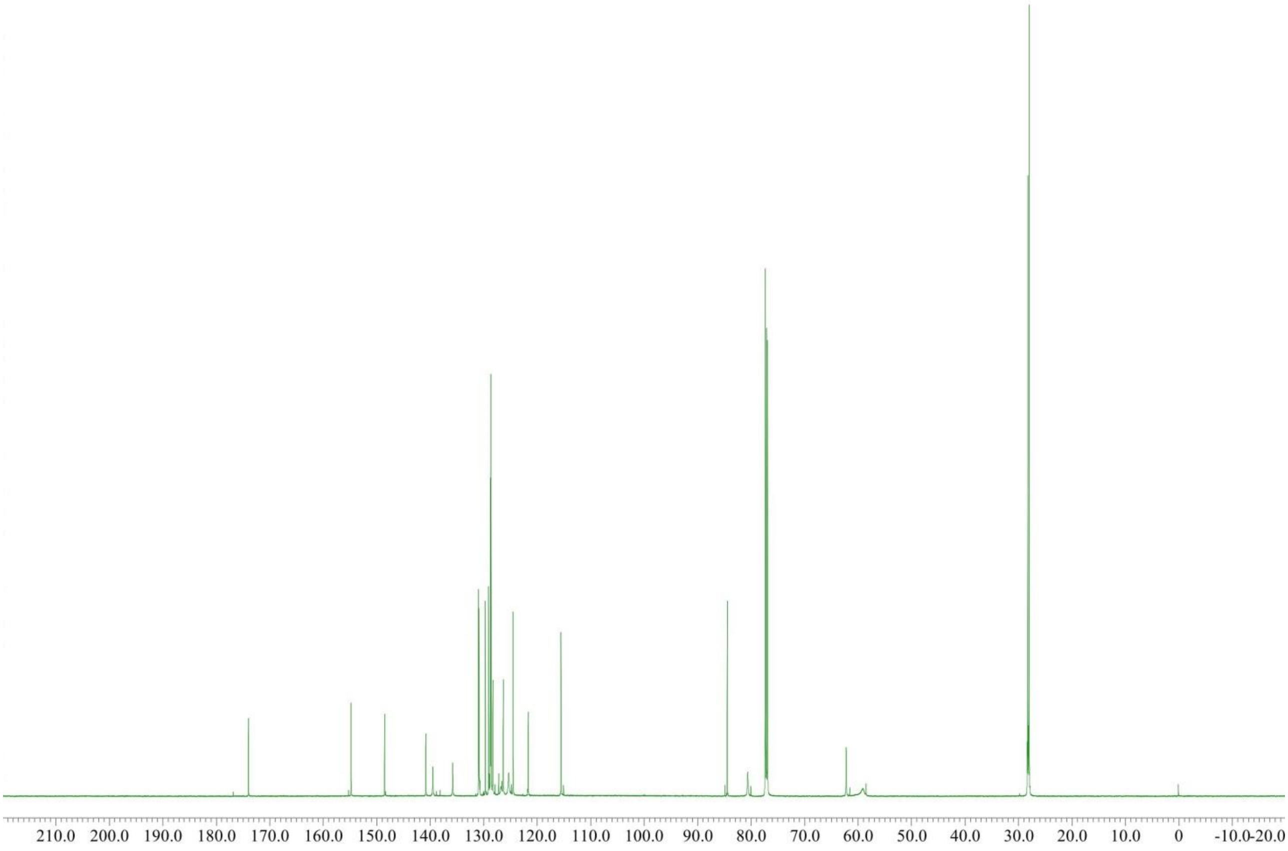
210.0 200.0 190.0 180.0 170.0 160.0 150.0 140.0 130.0 120.0 110.0 100.0 90.0 80.0 70.0 60.0 50.0 40.0 30.0 20.0 10.0 0 -10.0-20.0

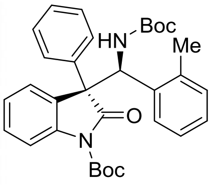
HN
Boc
Me
O
N
Boc

4af

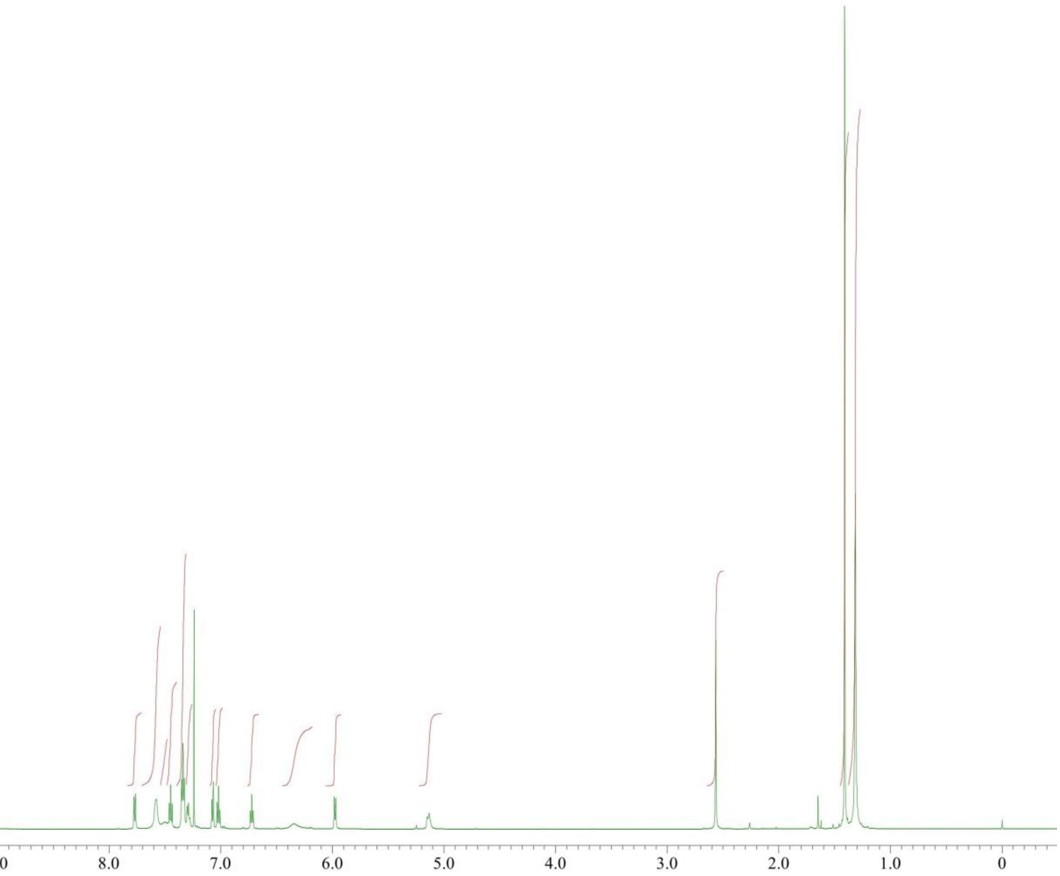
10.0 9.0 8.0 7.0 6.0 5.0 4.0 3.0 2.0 1.0 0

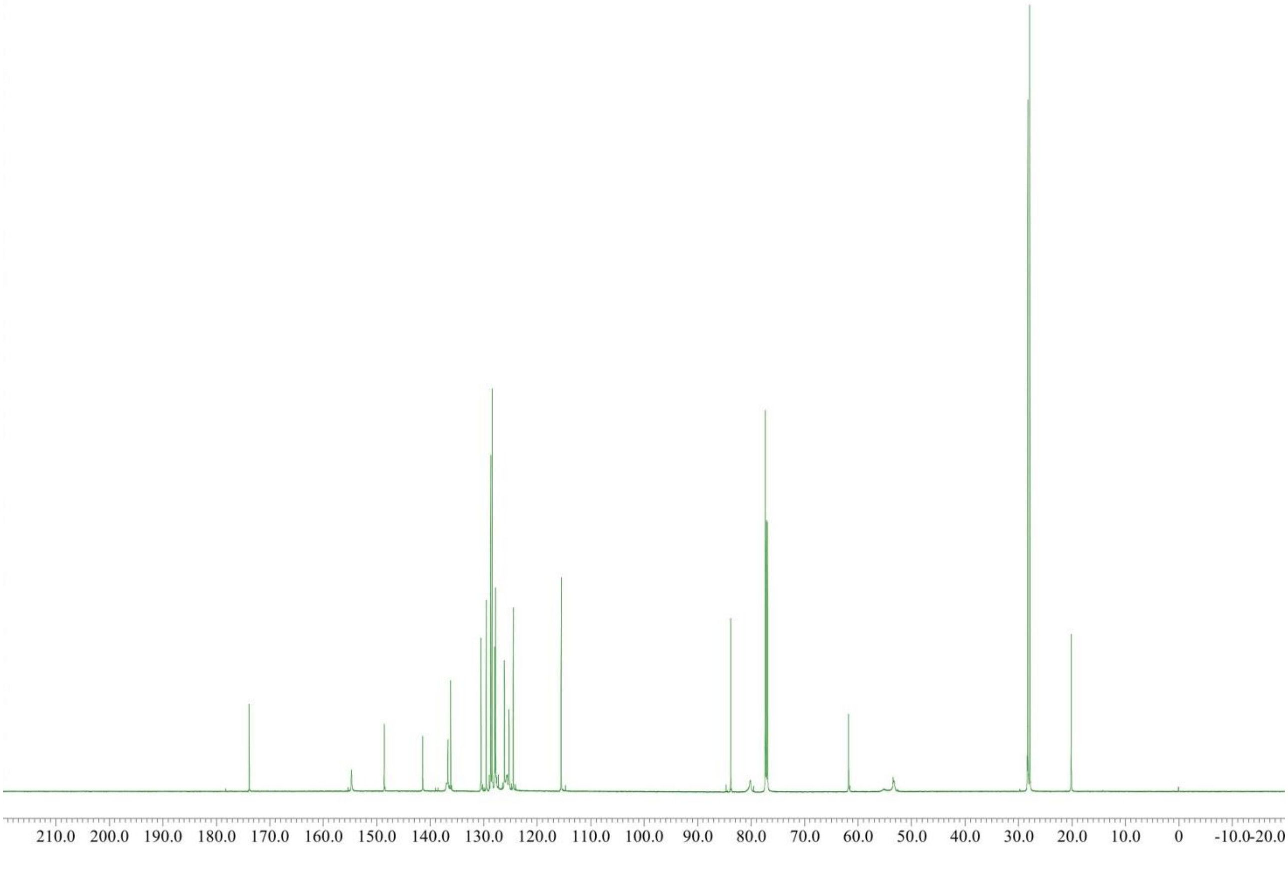
210.0 200.0 190.0 180.0 170.0 160.0 150.0 140.0 130.0 120.0 110.0 100.0 90.0 80.0 70.0 60.0 50.0 40.0 30.0 20.0 10.0 0 -10.0-20.0

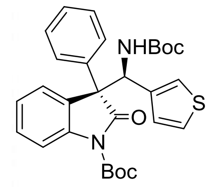
NHBoc
S
O
N
Boc

4ag

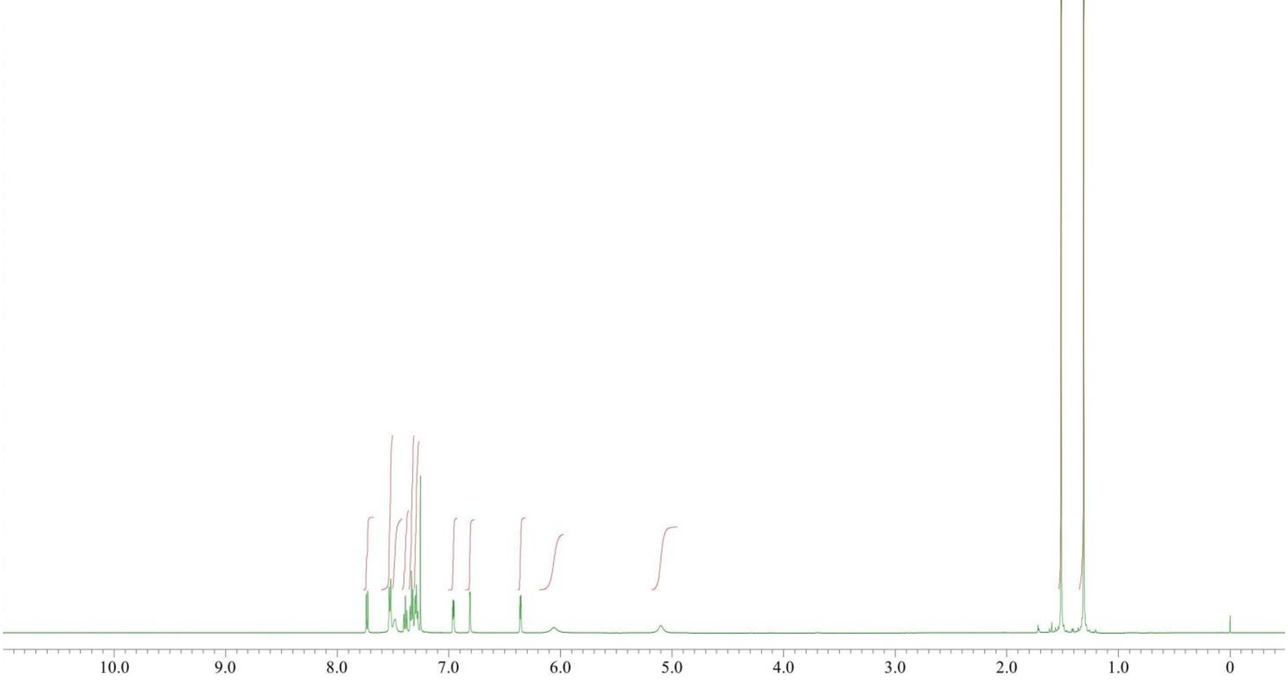
10.0 9.0 8.0 7.0 6.0 5.0 4.0 3.0 2.0 1.0 0

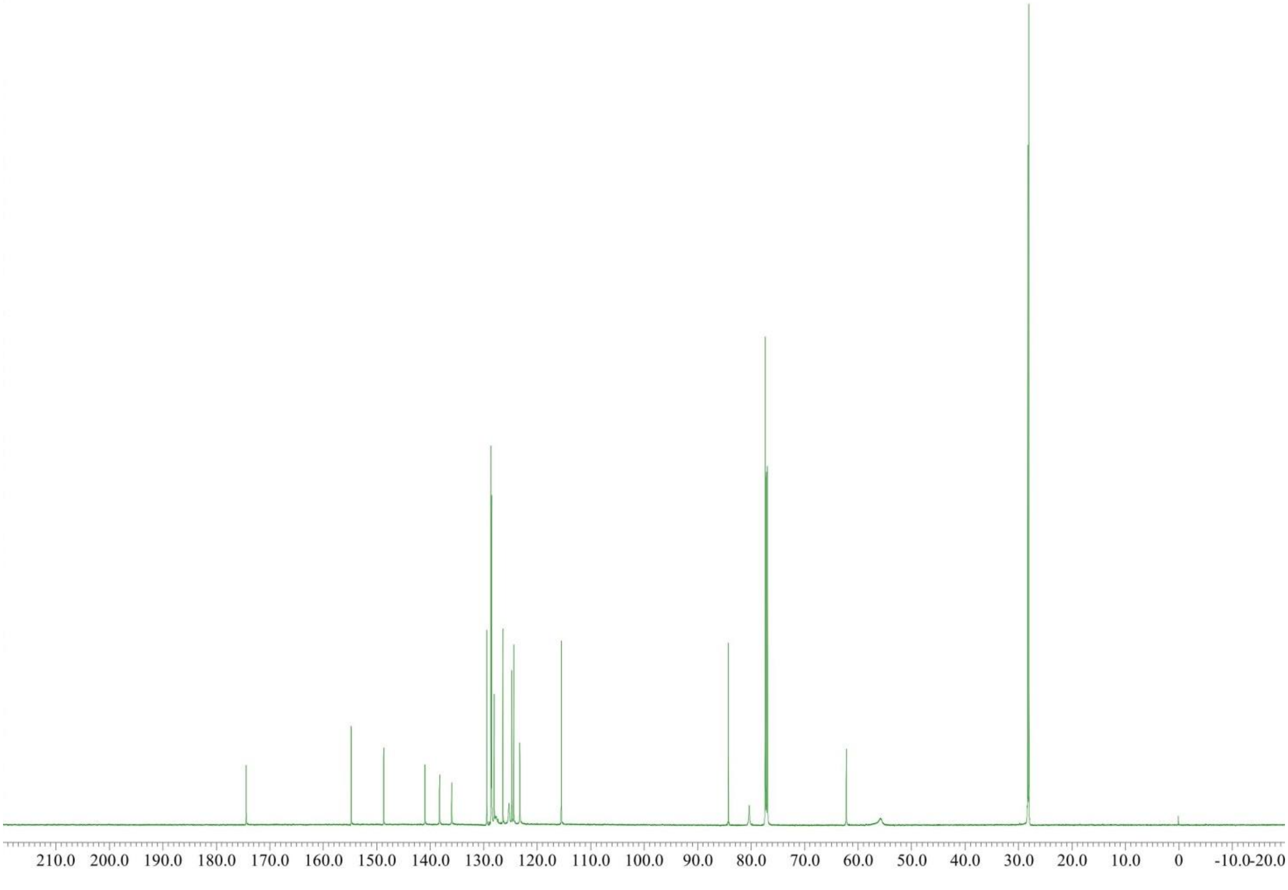
210.0 200.0 190.0 180.0 170.0 160.0 150.0 140.0 130.0 120.0 110.0 100.0 90.0 80.0 70.0 60.0 50.0 40.0 30.0 20.0 10.0 0 -10.0-20.0

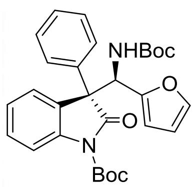
NHBoc
O
O
N
Boc
4ah

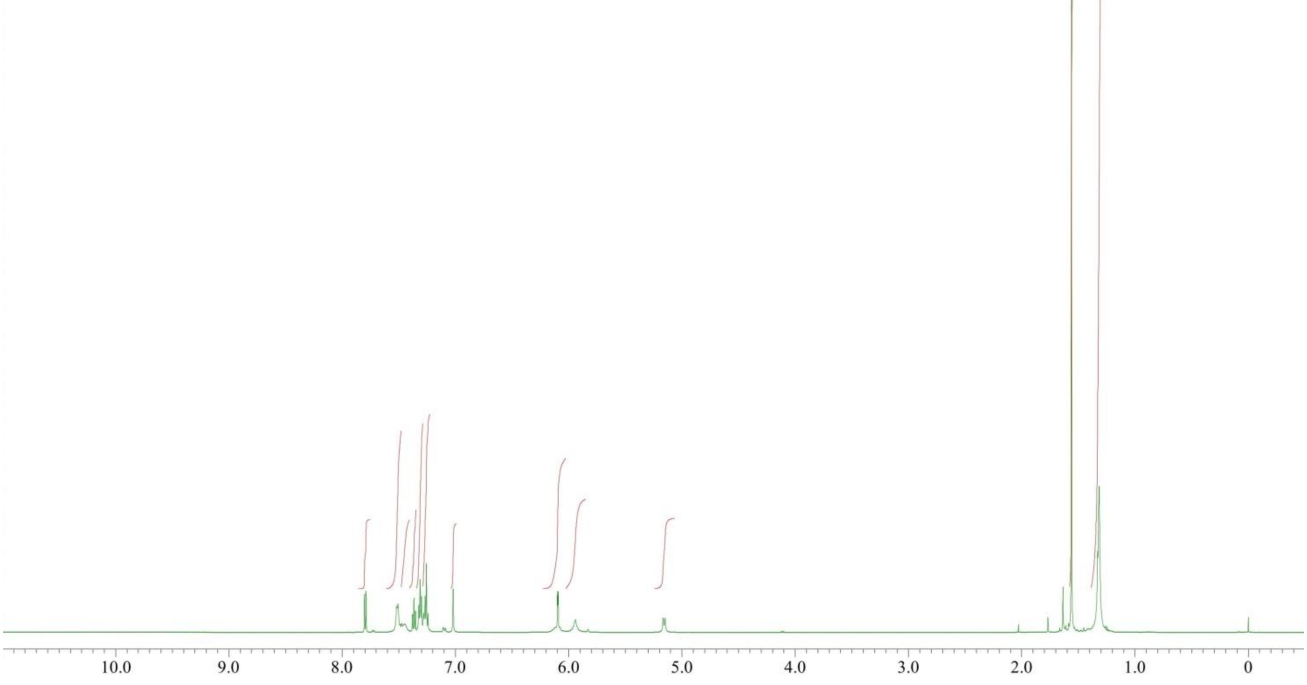
10.0 9.0 8.0 7.0 6.0 5.0 4.0 3.0 2.0 1.0 0

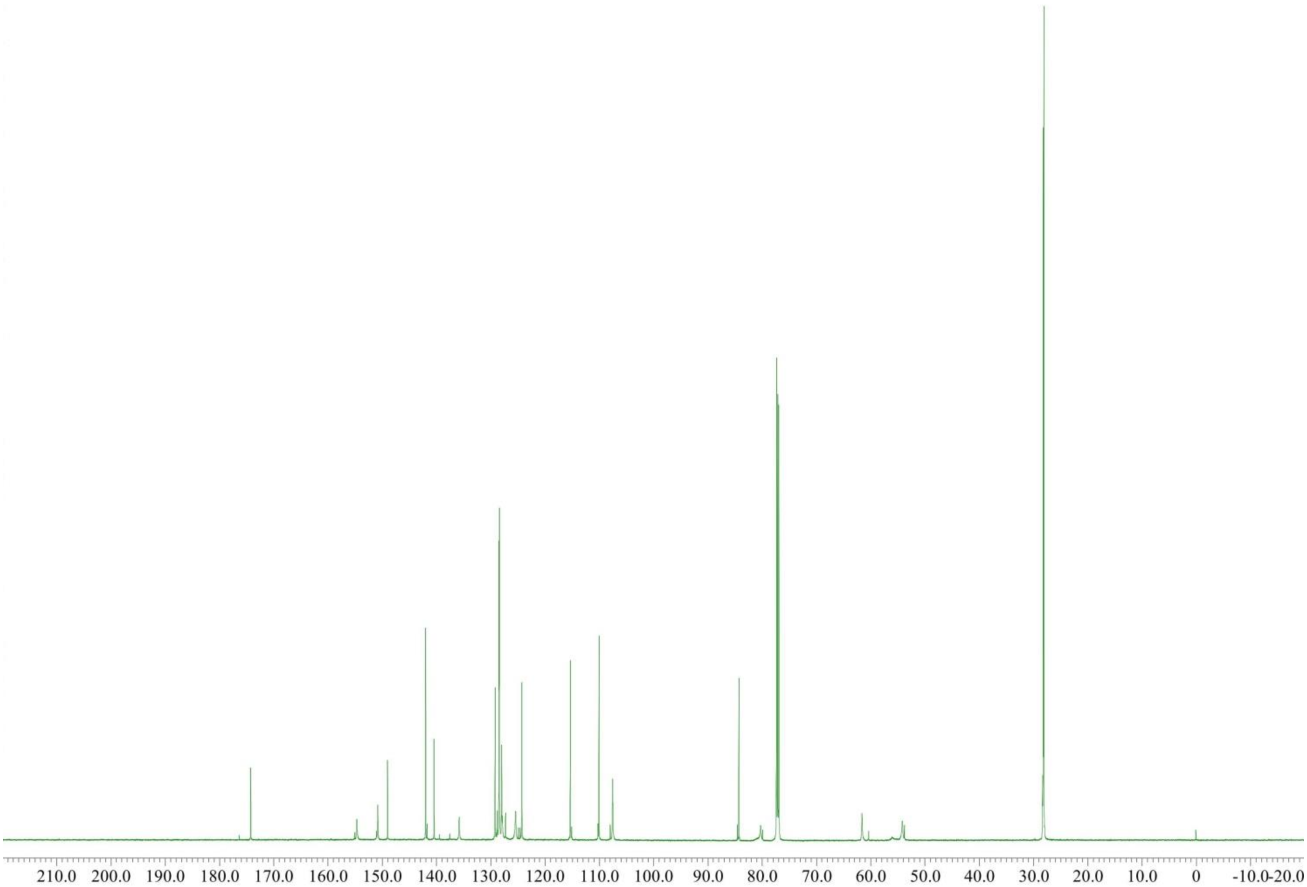
210.0 200.0 190.0 180.0 170.0 160.0 150.0 140.0 130.0 120.0 110.0 100.0 90.0 80.0 70.0 60.0 50.0 40.0 30.0 20.0 10.0 0 -10.0-20.0

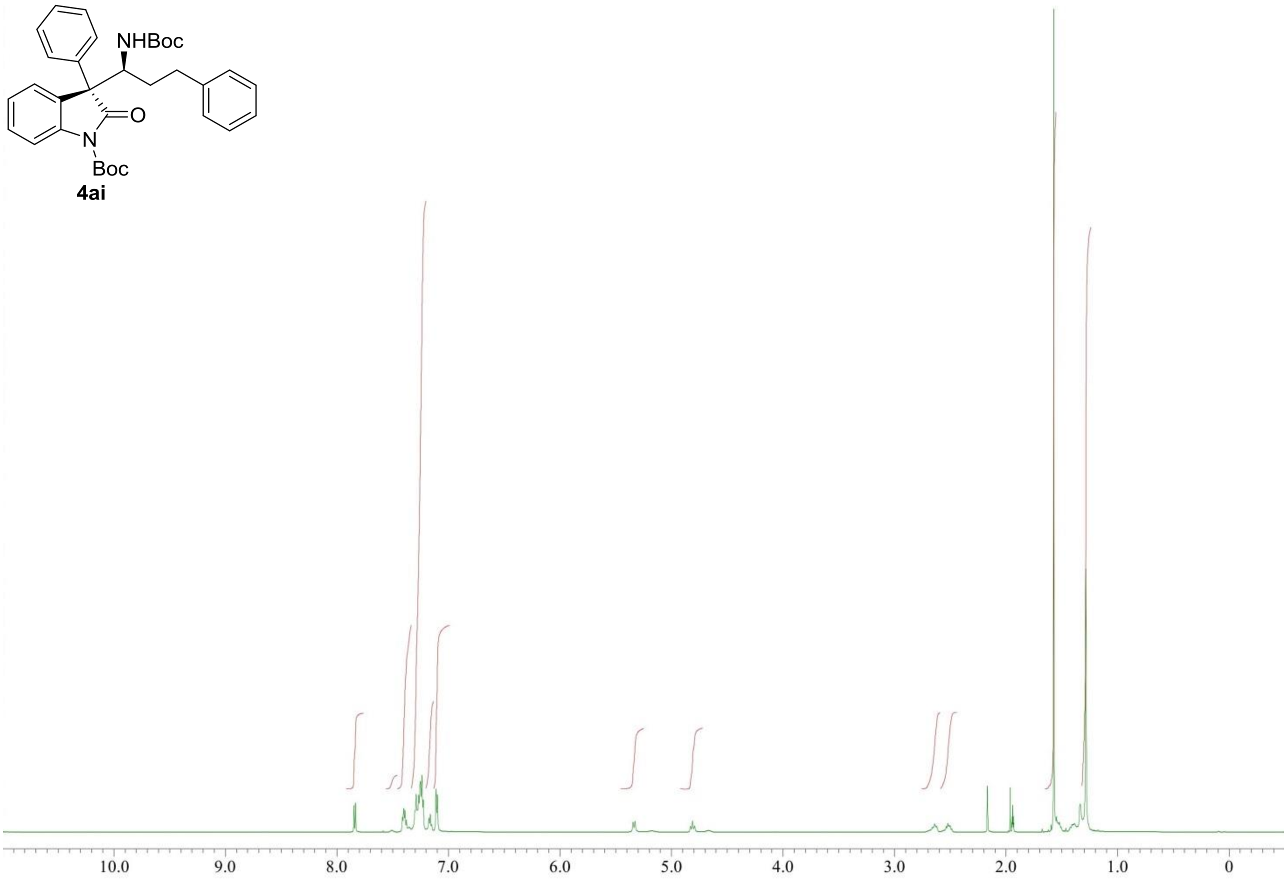
NHBoc
O
N
Boc

4ai

10.0 9.0 8.0 7.0 6.0 5.0 4.0 3.0 2.0 1.0 0

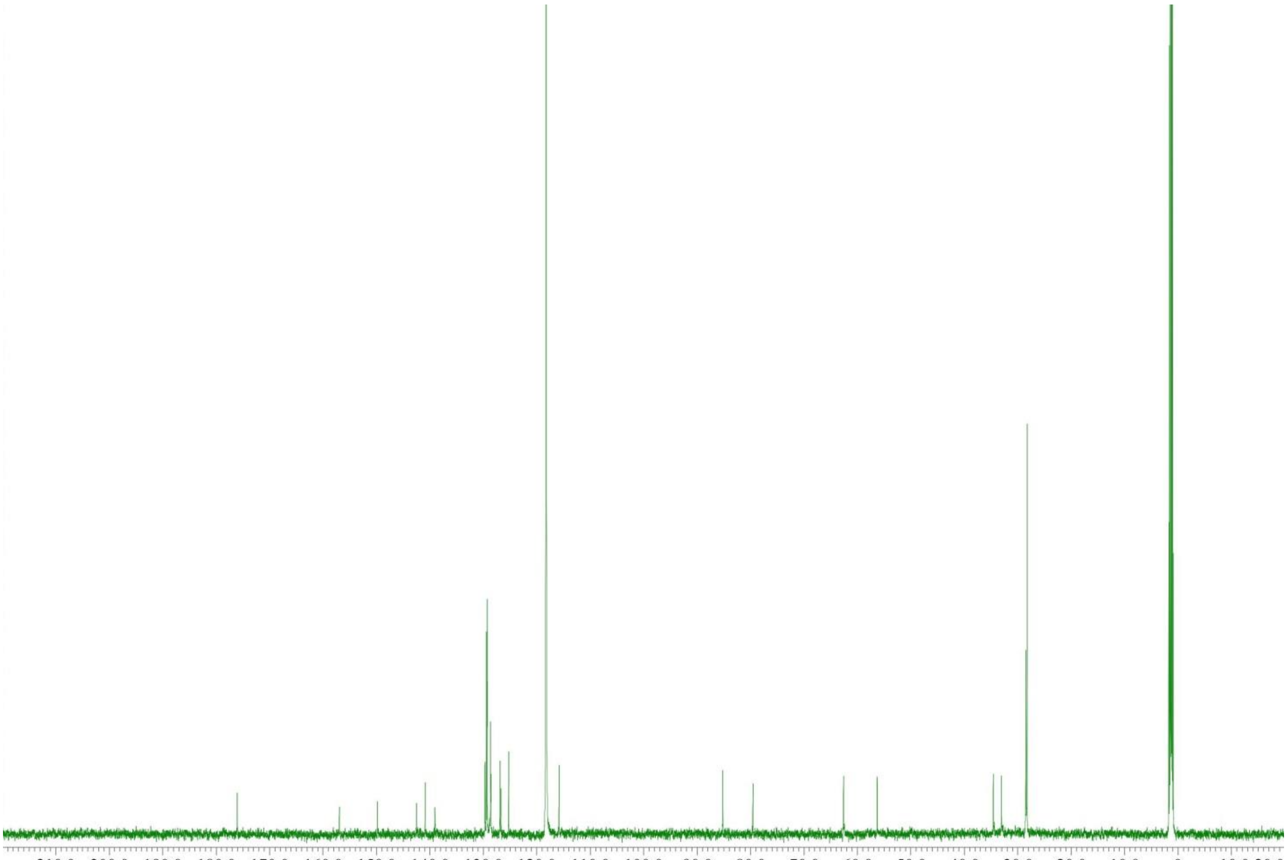
210.0 200.0 190.0 180.0 170.0 160.0 150.0 140.0 130.0 120.0 110.0 100.0 90.0 80.0 70.0 60.0 50.0 40.0 30.0 20.0 10.0 0 -10.0-20.0

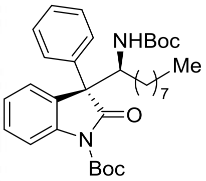
NHBoc
Me
7
O
N
Boc

4aj

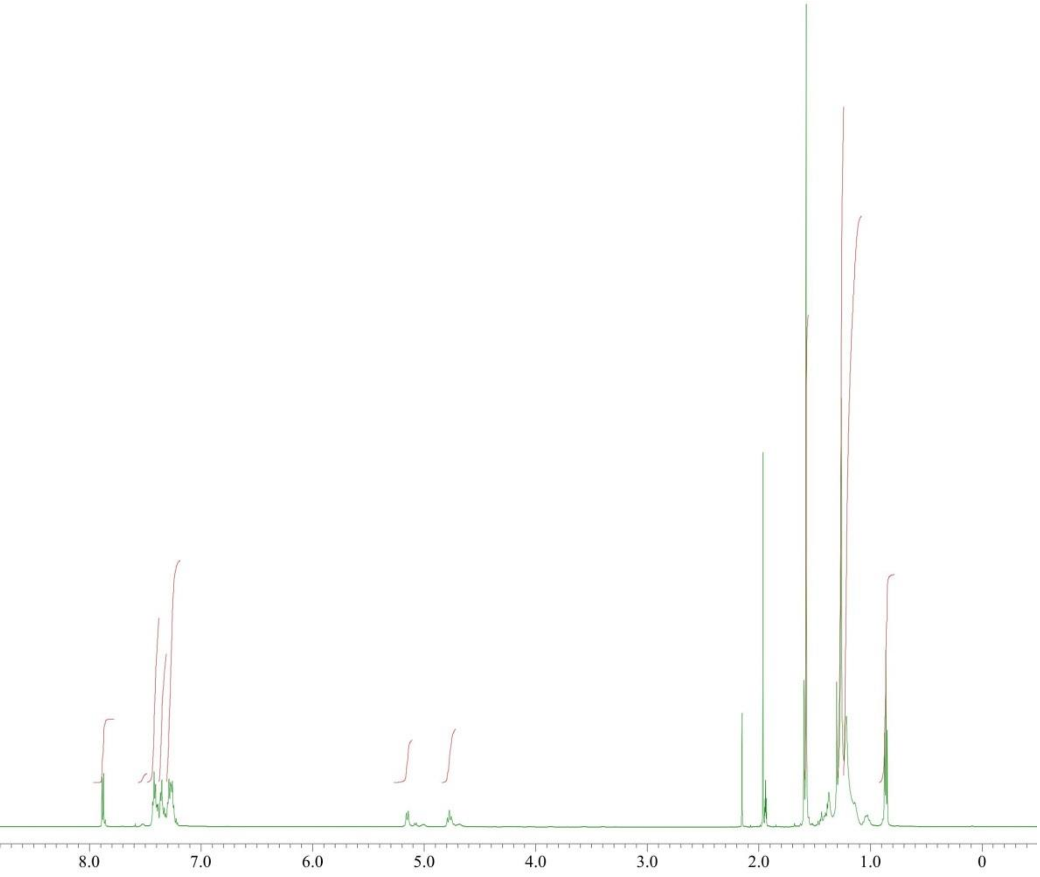
10.0 9.0 8.0 7.0 6.0 5.0 4.0 3.0 2.0 1.0 0

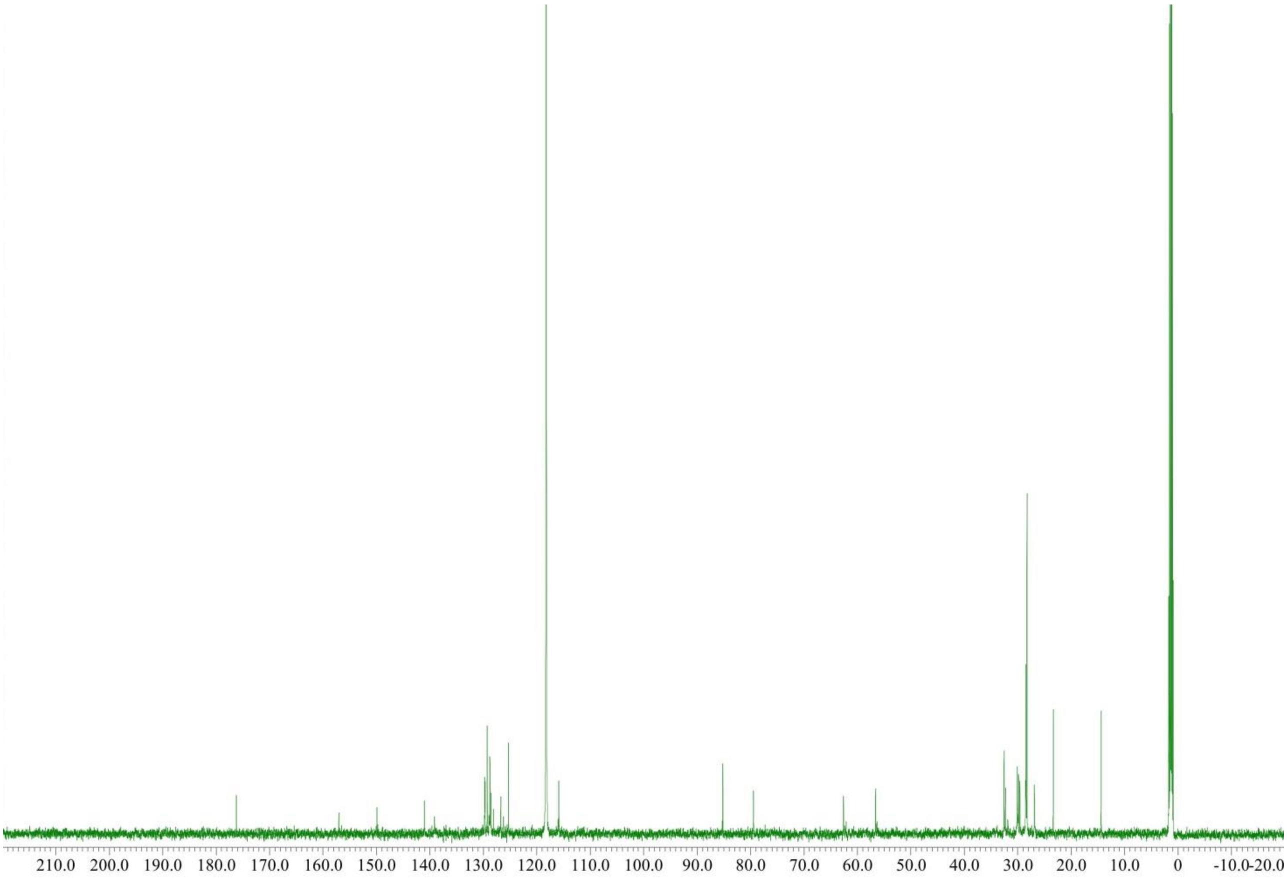
210.0 200.0 190.0 180.0 170.0 160.0 150.0 140.0 130.0 120.0 110.0 100.0 90.0 80.0 70.0 60.0 50.0 40.0 30.0 20.0 10.0 0 -10.0-20.0

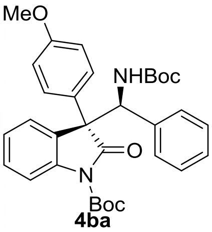
MeO
NHBoc
O
N
Boc

4ba

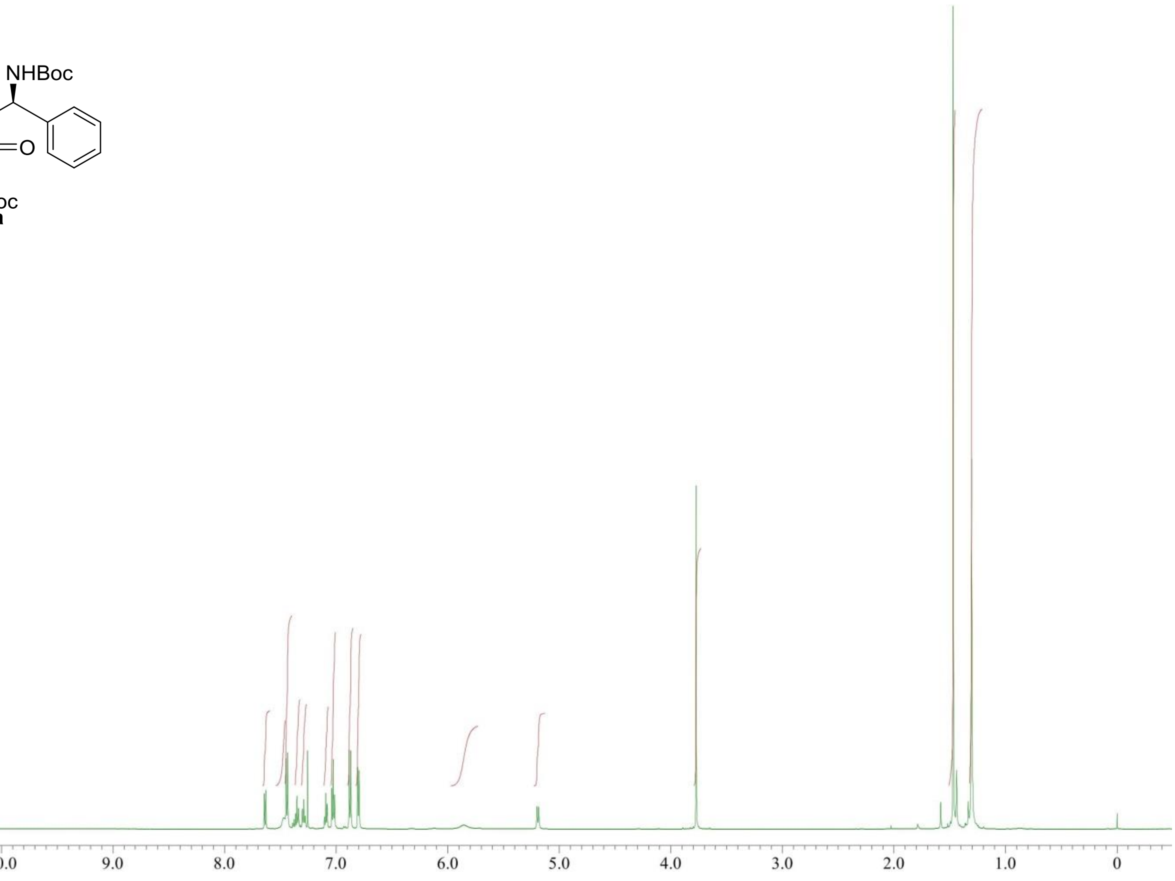
10.0 9.0 8.0 7.0 6.0 5.0 4.0 3.0 2.0 1.0 0

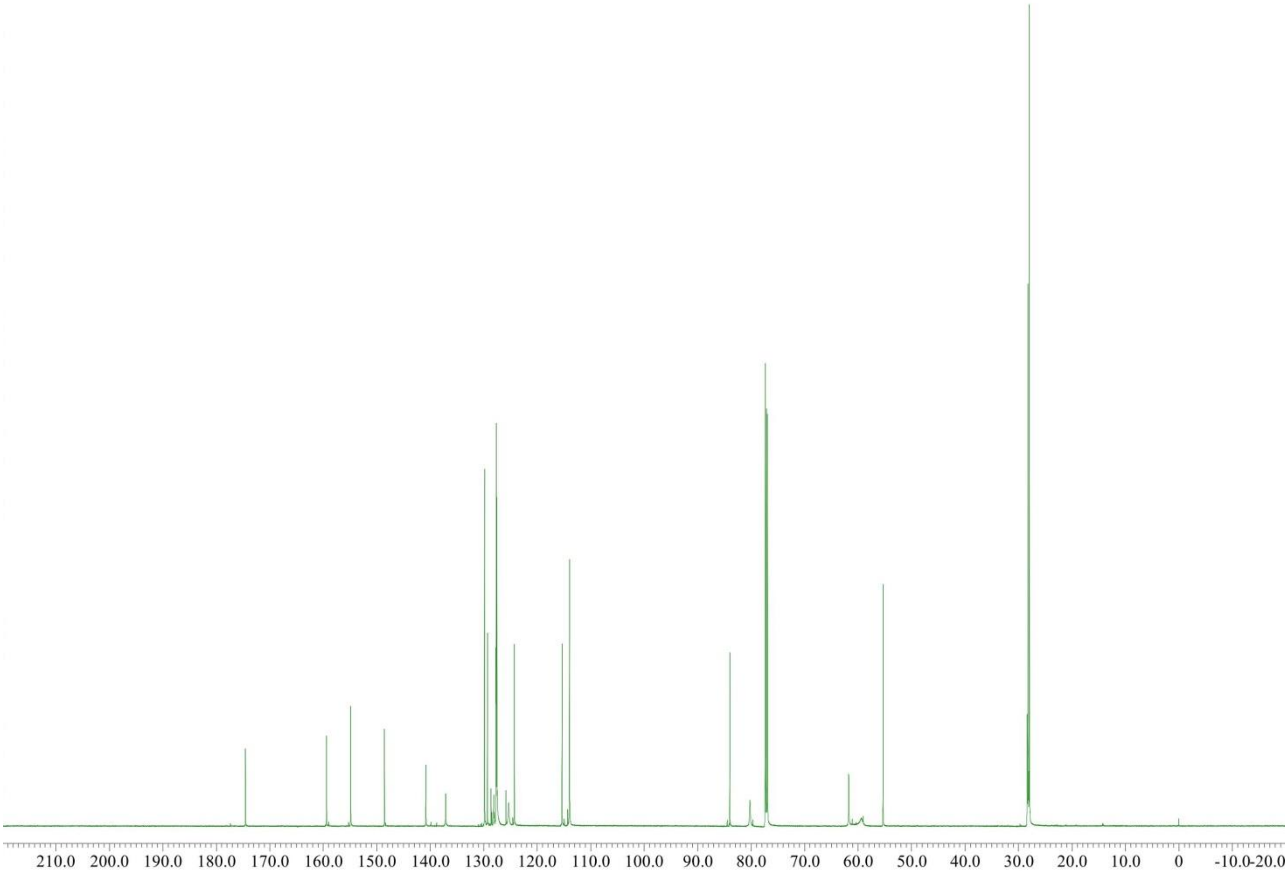
210.0 200.0 190.0 180.0 170.0 160.0 150.0 140.0 130.0 120.0 110.0 100.0 90.0 80.0 70.0 60.0 50.0 40.0 30.0 20.0 10.0 0 -10.0-20.0

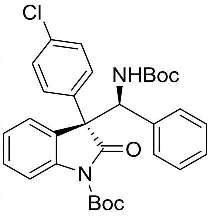
Cl
NHBoc
O
N
Boc

4ca

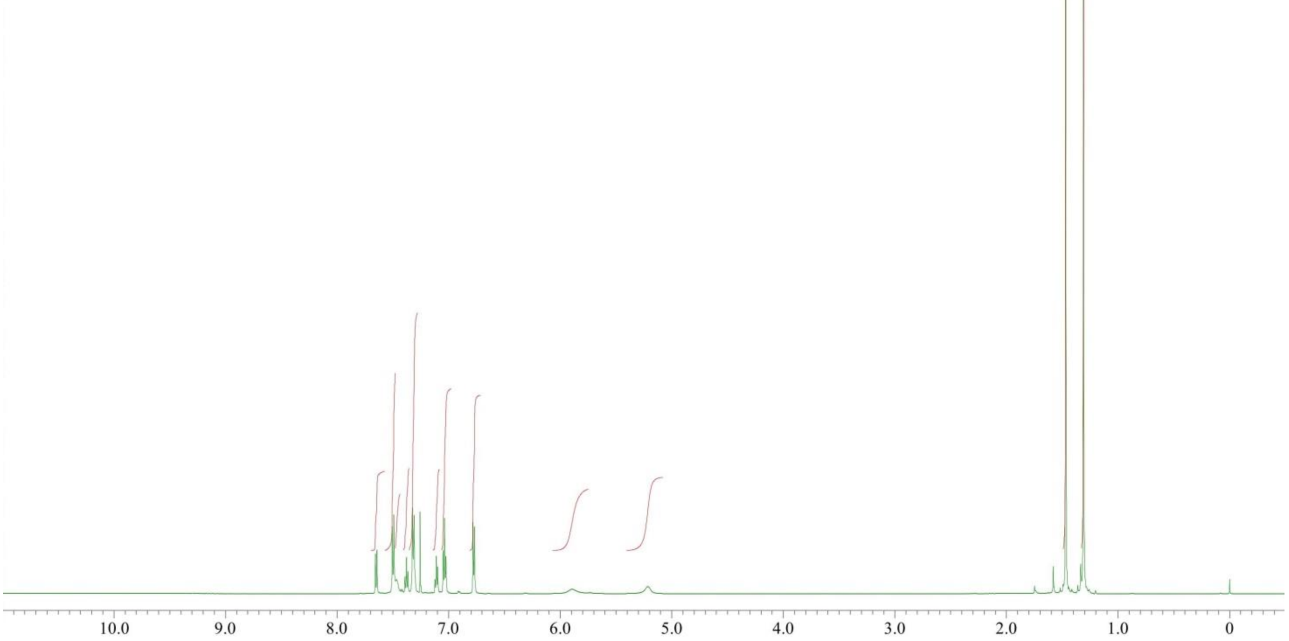
10.0
9.0
8.0
7.0
6.0
5.0
4.0
3.0
2.0
1.0
0

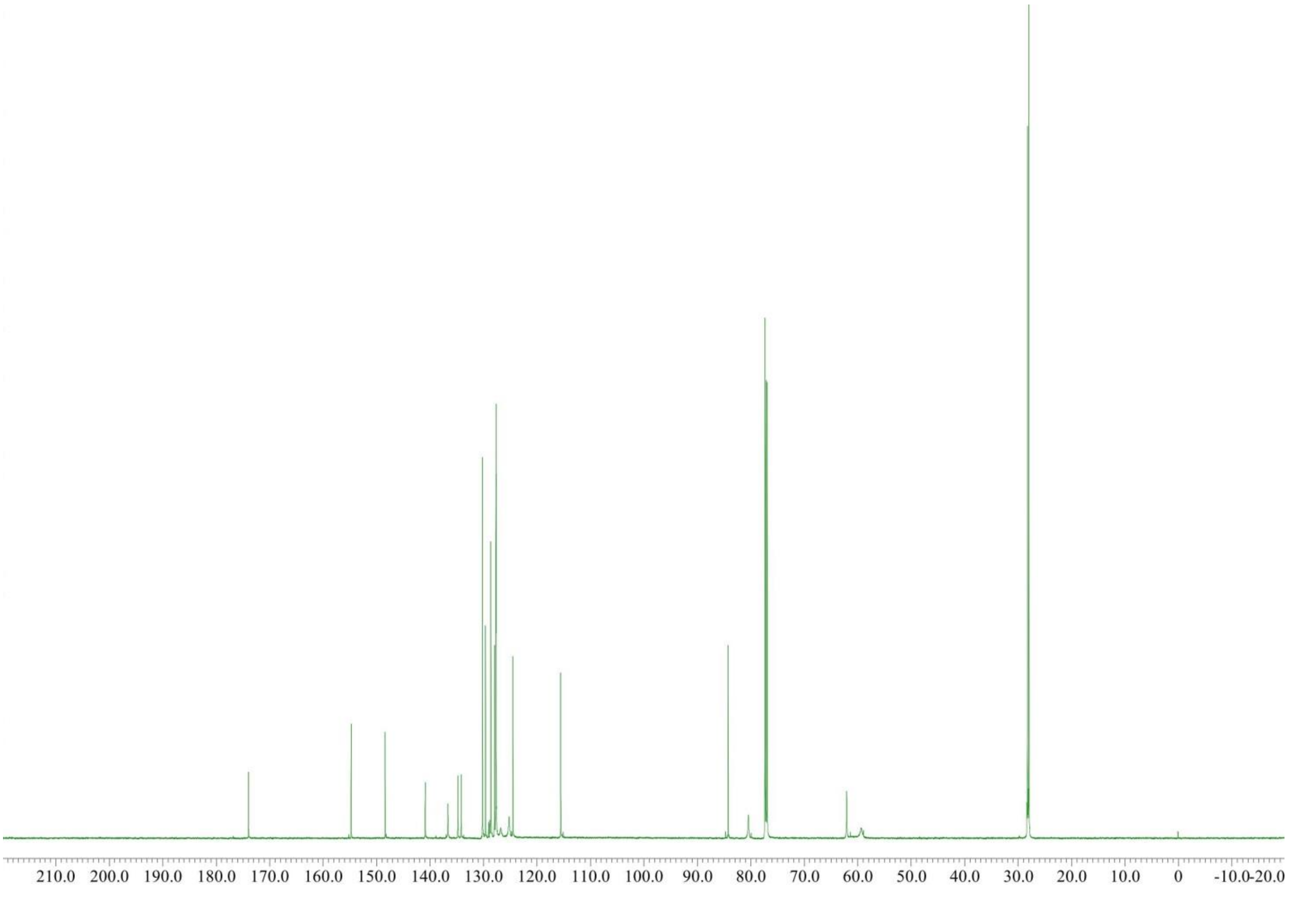
210.0
200.0
190.0
180.0
170.0
160.0
150.0
140.0
130.0
120.0
110.0
100.0
90.0
80.0
70.0
60.0
50.0
40.0
30.0
20.0
10.0
0
-10.0-20.0

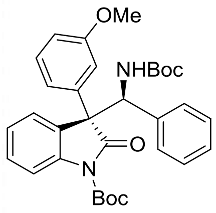
OMe
NHBoc
O
N
Boc

4da

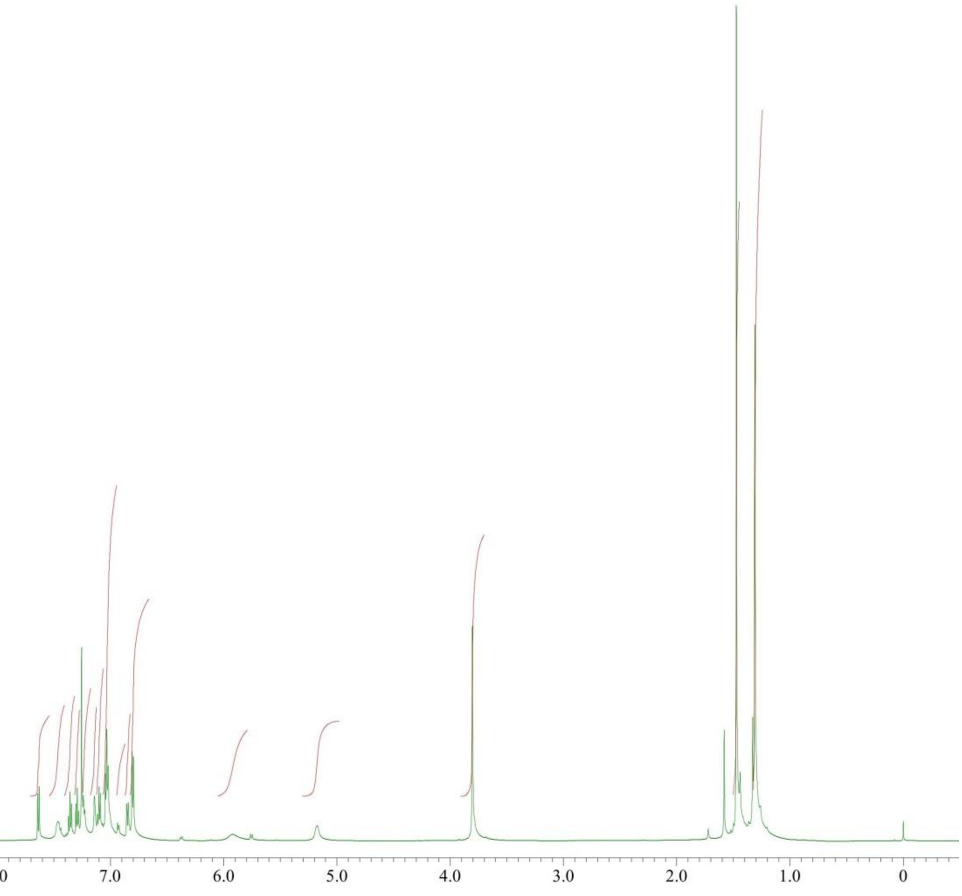
10.0 9.0 8.0 7.0 6.0 5.0 4.0 3.0 2.0 1.0 0

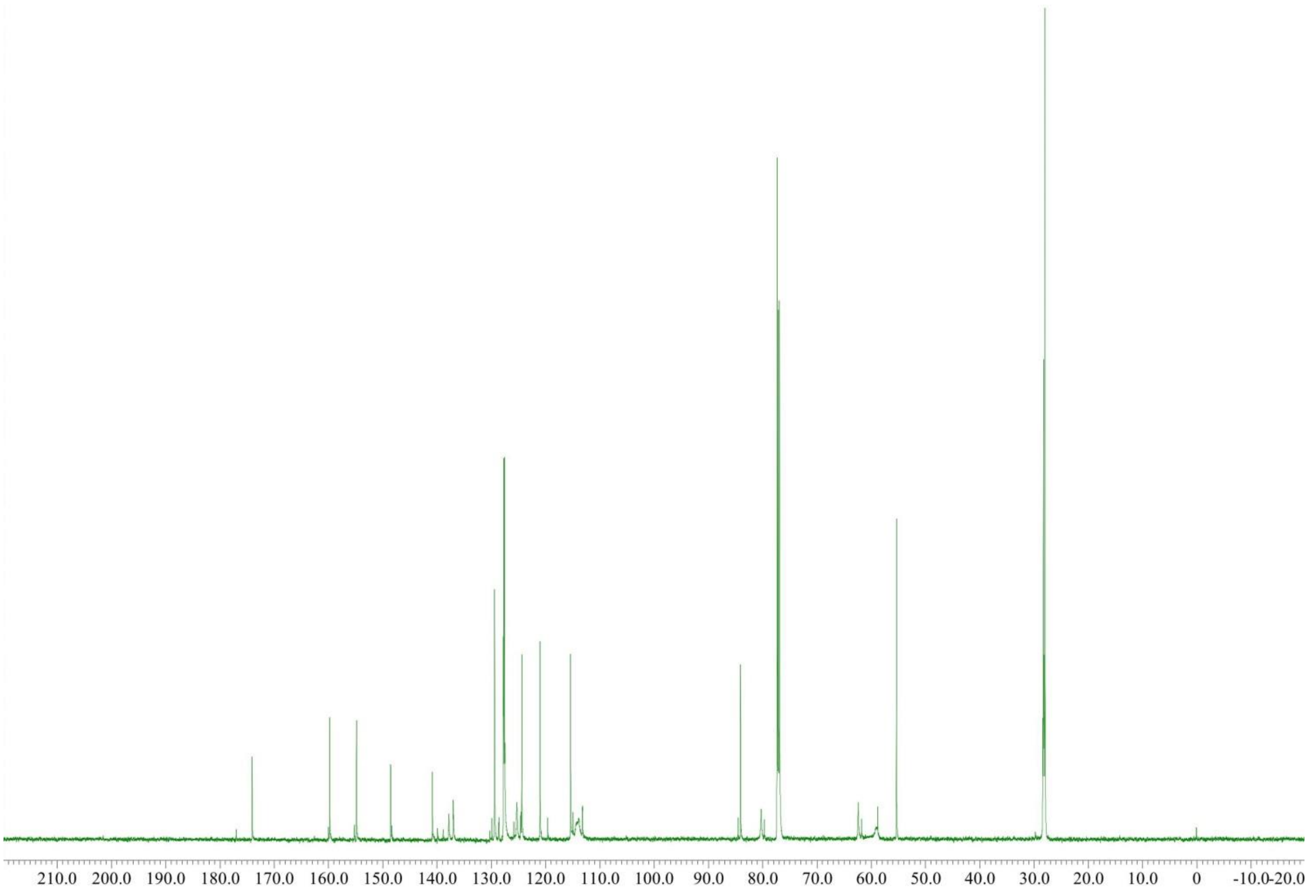
210.0 200.0 190.0 180.0 170.0 160.0 150.0 140.0 130.0 120.0 110.0 100.0 90.0 80.0 70.0 60.0 50.0 40.0 30.0 20.0 10.0 0 -10.0-20.0

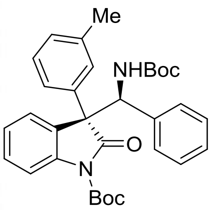
Me
NHBoc
O
N
Boc

4ea

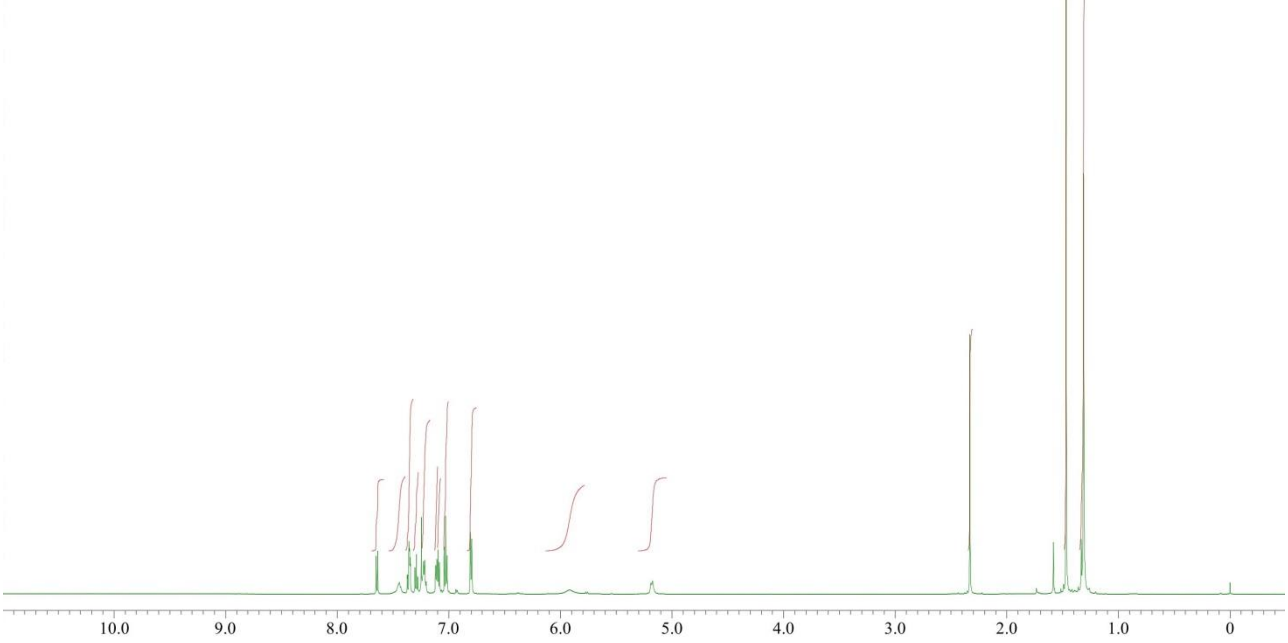
10.0 9.0 8.0 7.0 6.0 5.0 4.0 3.0 2.0 1.0 0

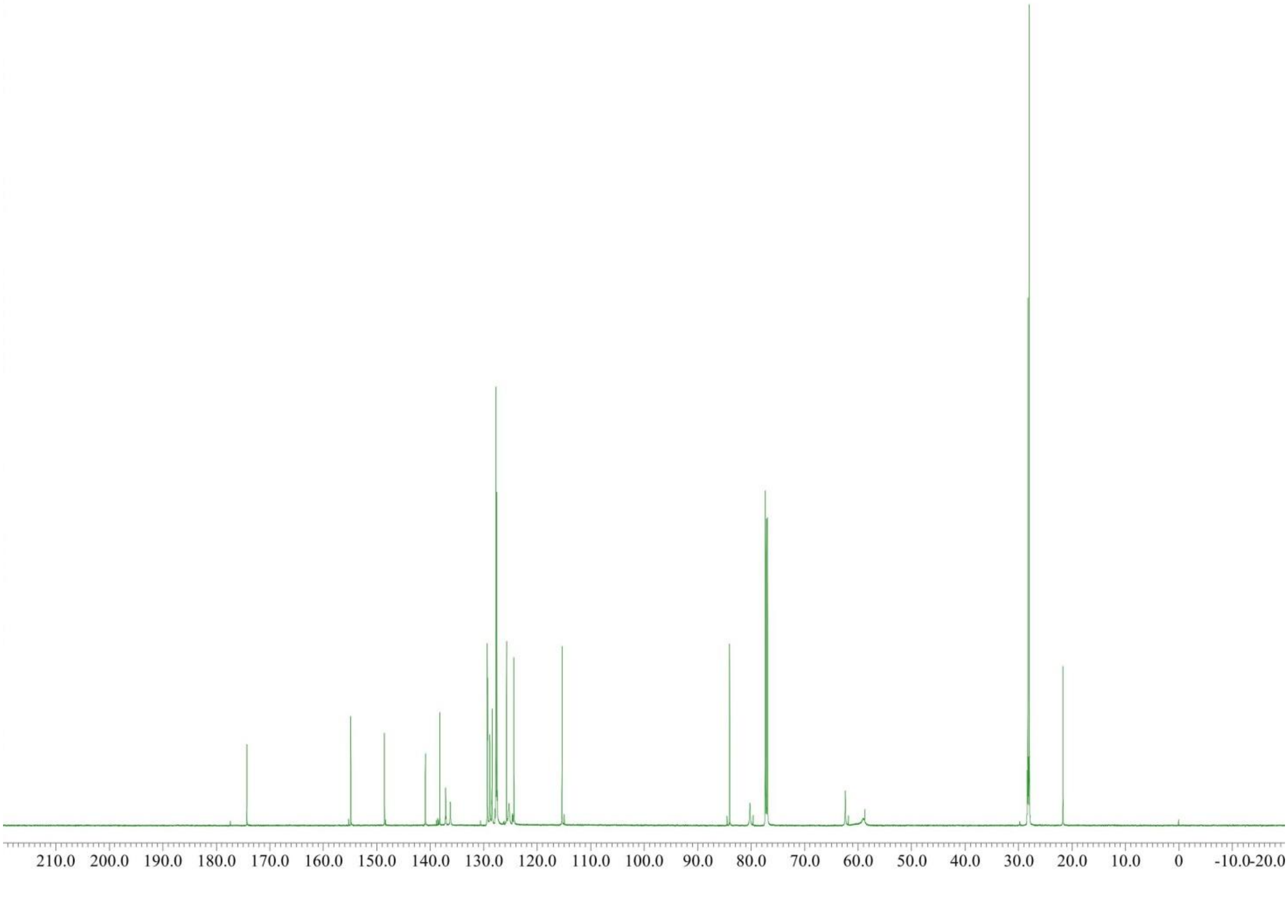
210.0 200.0 190.0 180.0 170.0 160.0 150.0 140.0 130.0 120.0 110.0 100.0 90.0 80.0 70.0 60.0 50.0 40.0 30.0 20.0 10.0 0 -10.0-20.0

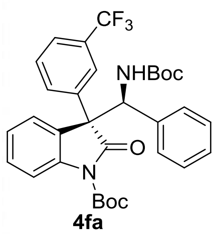
CF$_3$
NHBoc
O
N
Boc

4fa

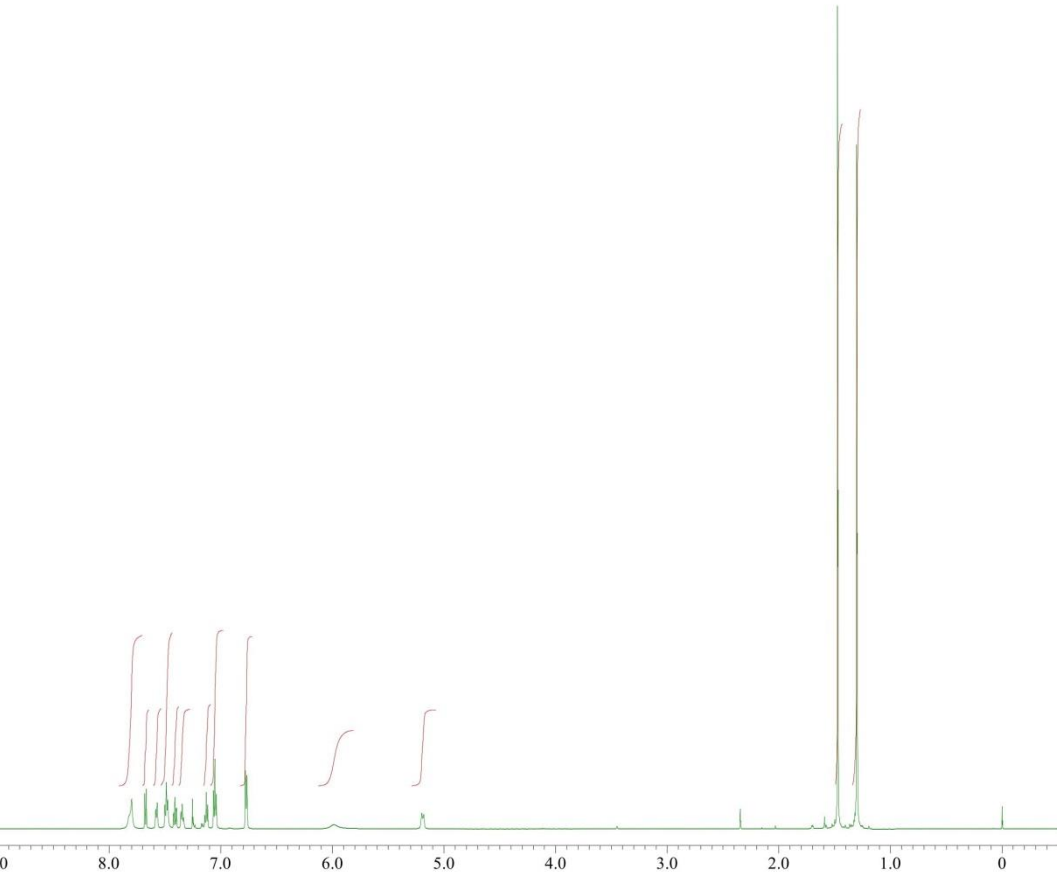
10.0 9.0 8.0 7.0 6.0 5.0 4.0 3.0 2.0 1.0 0

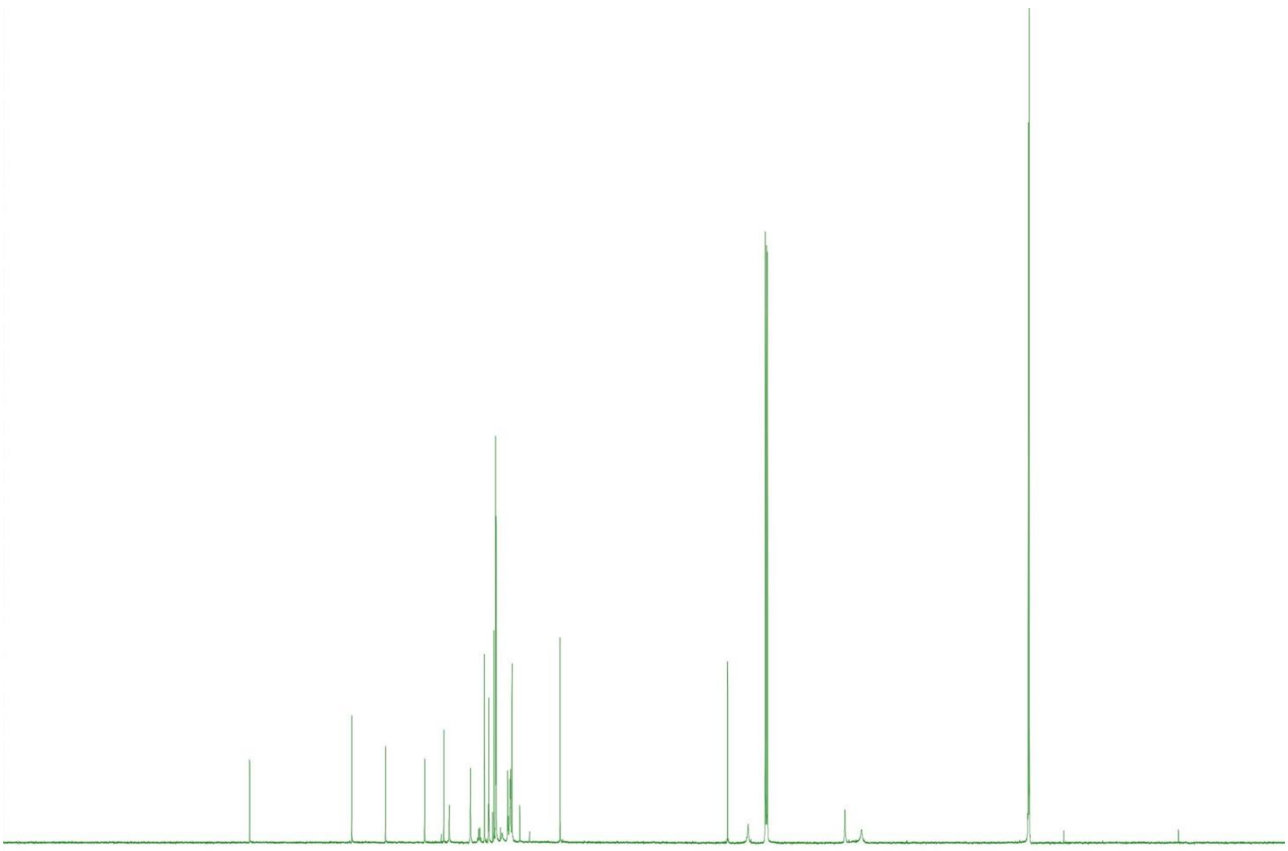
210.0 200.0 190.0 180.0 170.0 160.0 150.0 140.0 130.0 120.0 110.0 100.0 90.0 80.0 70.0 60.0 50.0 40.0 30.0 20.0 10.0 0 -10.0-20.0

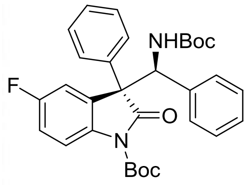
NHBoc
F
O
N
Boc

4ga

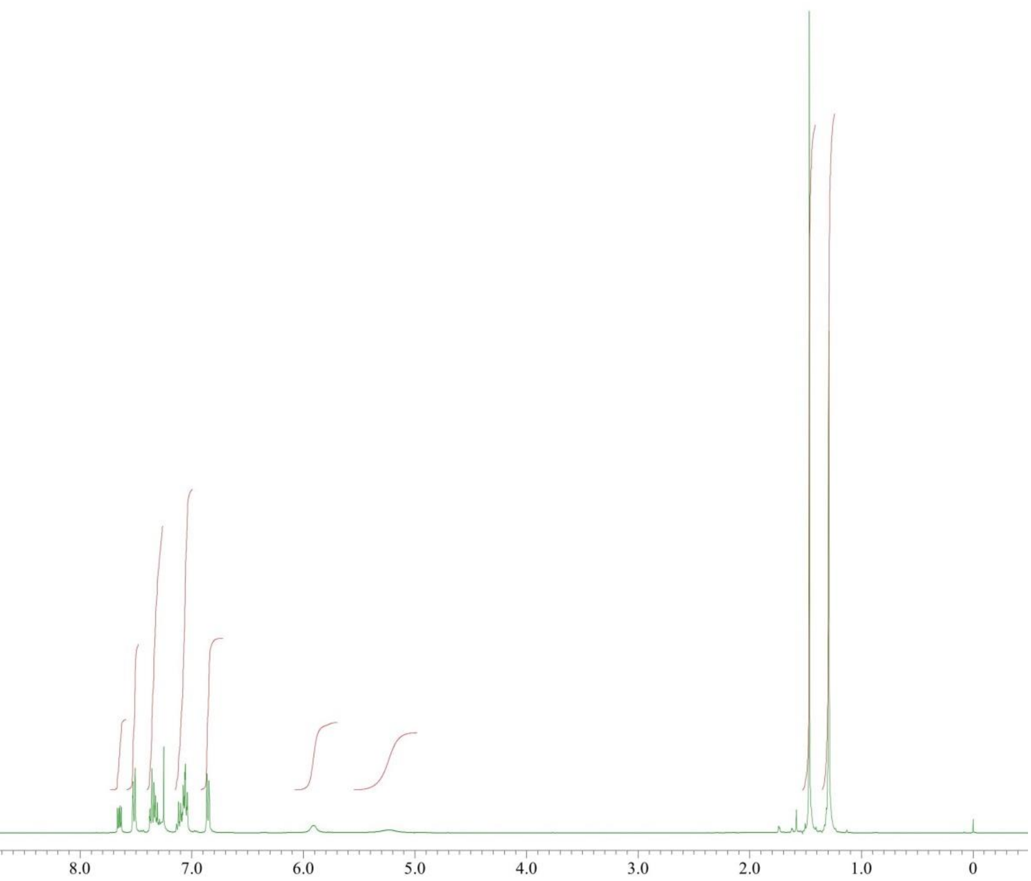
11.0 10.0 9.0 8.0 7.0 6.0 5.0 4.0 3.0 2.0 1.0 0

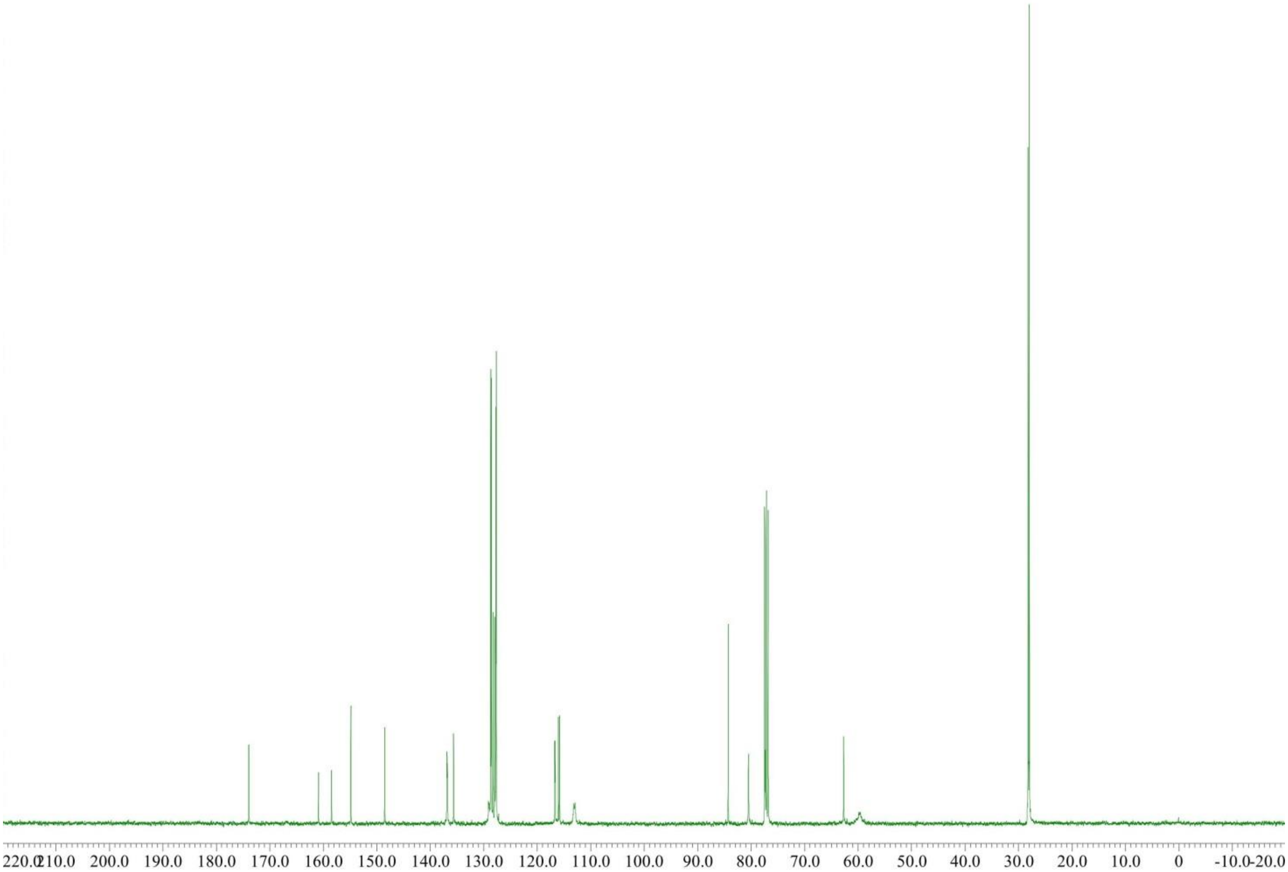
220.0 210.0 200.0 190.0 180.0 170.0 160.0 150.0 140.0 130.0 120.0 110.0 100.0 90.0 80.0 70.0 60.0 50.0 40.0 30.0 20.0 10.0 0 -10.0 -20.0

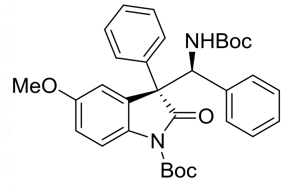
NHBoc
MeO
O
N
Boc

4ha

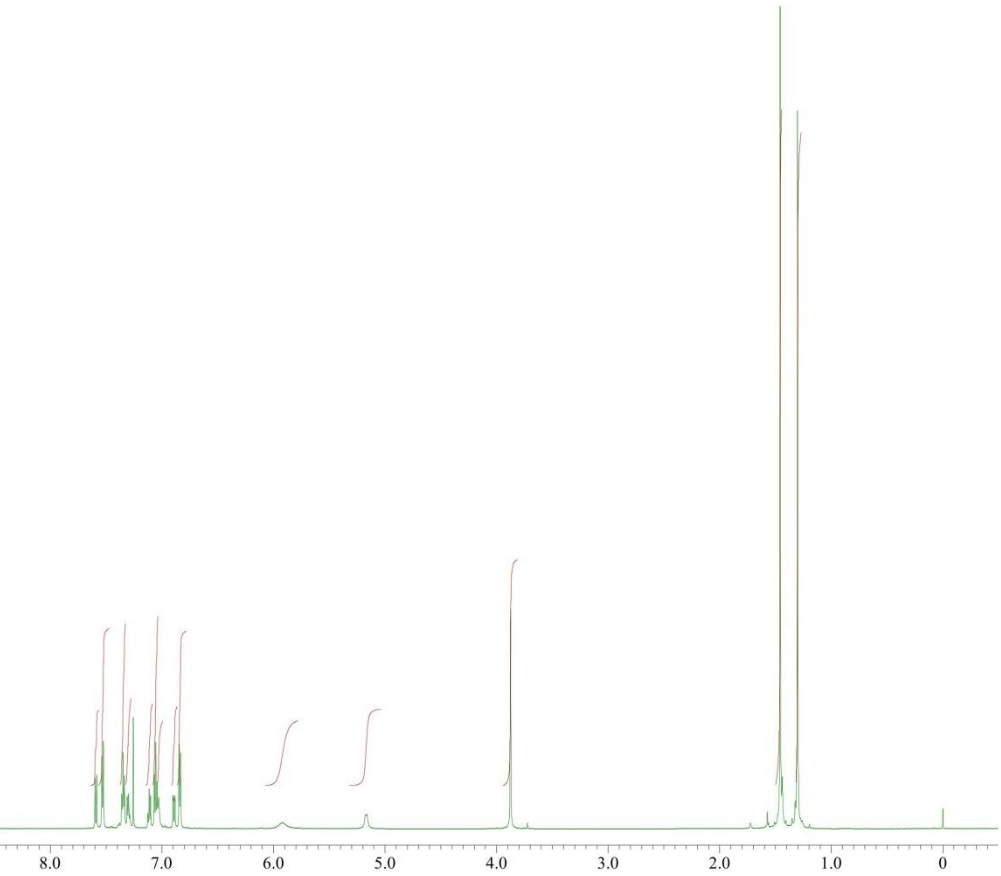
10.0 9.0 8.0 7.0 6.0 5.0 4.0 3.0 2.0 1.0 0

210.0 200.0 190.0 180.0 170.0 160.0 150.0 140.0 130.0 120.0 110.0 100.0 90.0 80.0 70.0 60.0 50.0 40.0 30.0 20.0 10.0 0 -10.0-20.0

HPLC traces:

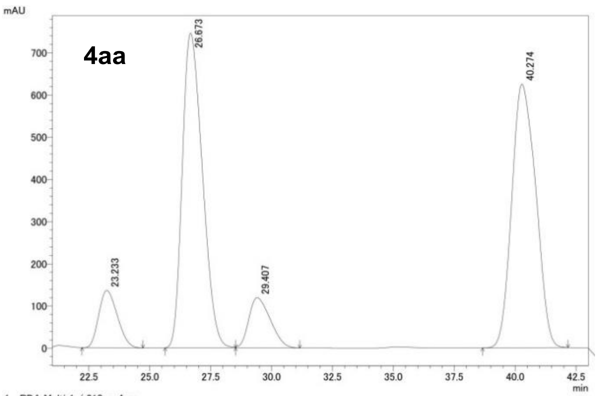

1 PDA Multi 1 / 210nm,4nm

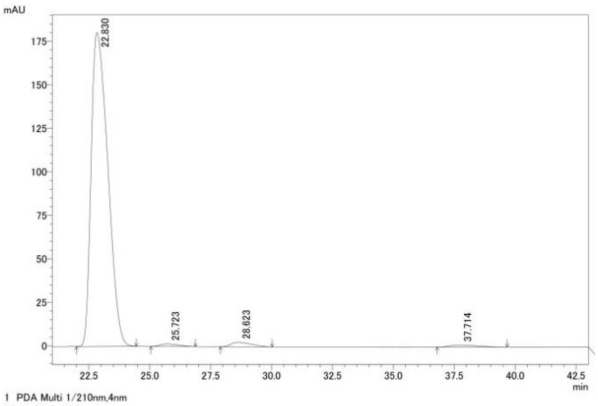

1 PDA Multi 1/210nm,4nm

PDA Ch1 210nm

ピーク#	保持時間	面積%
1	22.830	96.519
2	25.723	0.866
3	28.623	1.643
4	37.714	0.972
合計		100.000

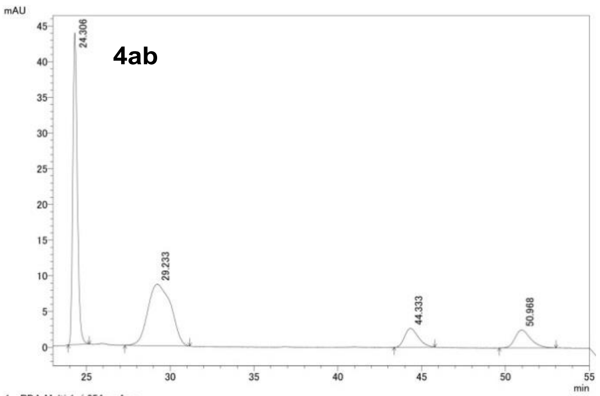

1 PDA Multi 1 / 254nm,4nm

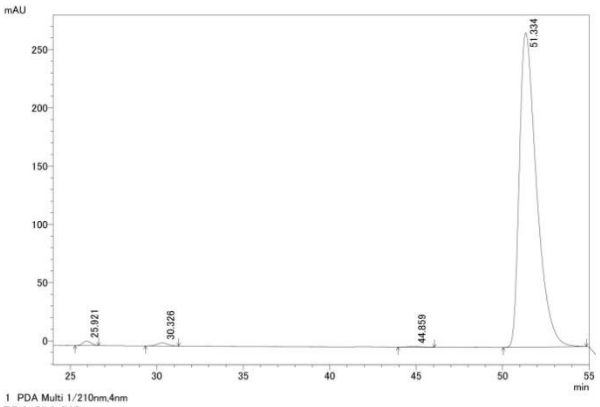

1 PDA Multi 1/210nm,4nm

PDA Ch1 210nm

ピーク#	保持時間	面積%
1	25.921	0.664
2	30.326	0.637
3	44.859	0.155
4	51.334	98.544
合計		100.000

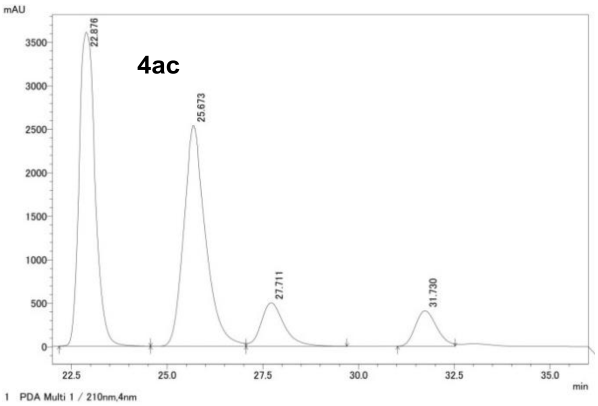

1 PDA Multi 1 / 210nm,4nm

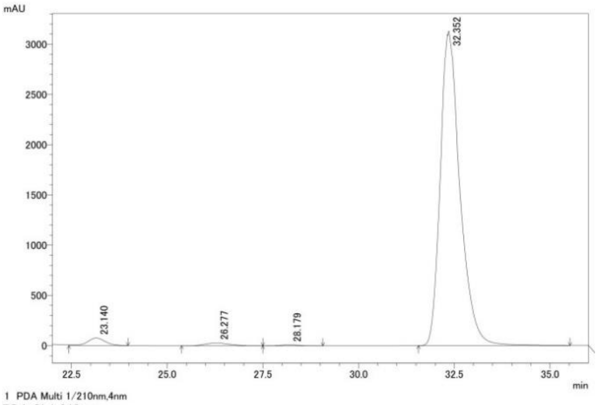

1 PDA Multi 1/210nm,4nm

PDA Ch1 210nm

ピーク#	保持時間	面積%
1	23.140	2.167
2	26.277	1.047
3	28.179	0.244
4	32.352	96.542
合計		100.000

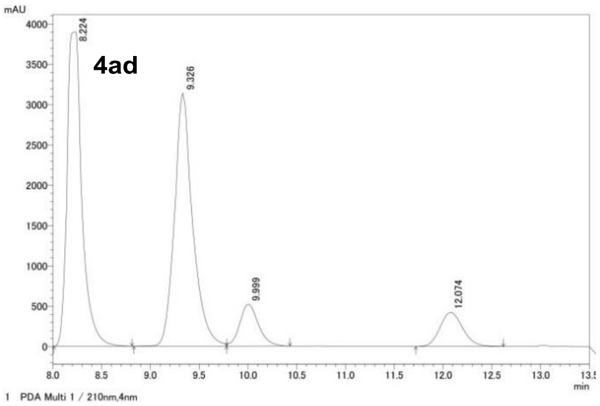

1 PDA Multi 1 / 210nm,4nm

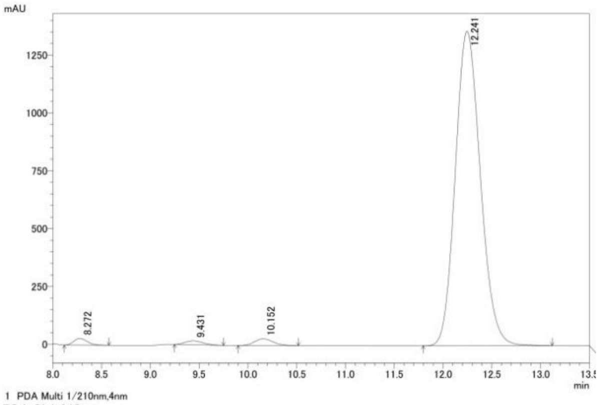

1 PDA Multi 1/210nm,4nm

PDA Ch1 210nm

ピーク#	保持時間	面積%
1	8.272	1.127
2	9.431	0.873
3	10.152	1.519
4	12.241	96.480
合計		100.000

4ae

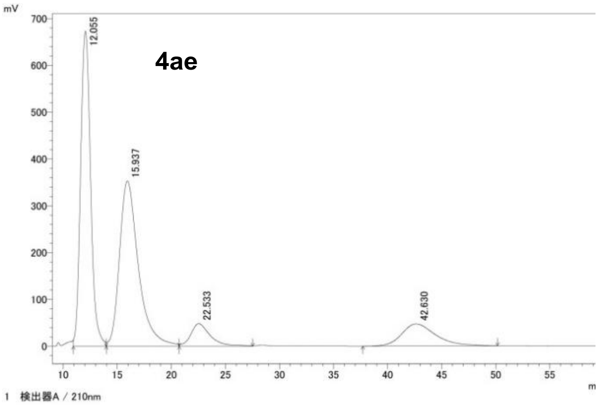

4af

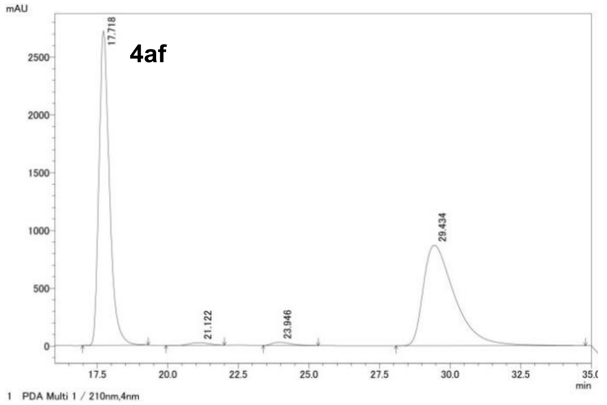

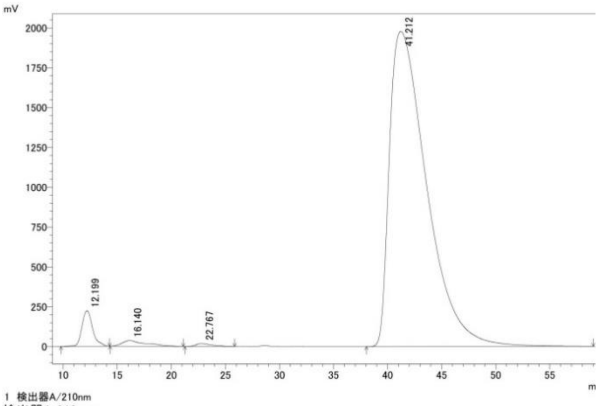

検出器A 210nm

ピーク#	保持時間	面積%
1	12.199	3.091
2	16.140	1.091
3	22.767	0.375
4	41.212	95.442
合計		100.000

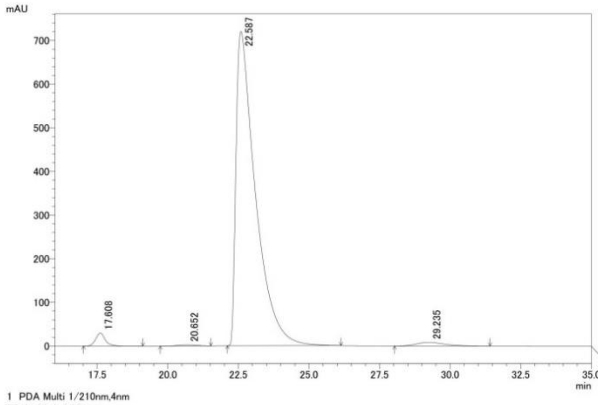

PDA Ch1 210nm

ピーク#	保持時間	面積%
1	17.608	1.983
2	20.652	0.307
3	22.587	96.112
4	29.235	1.598
合計		100.000

4ag

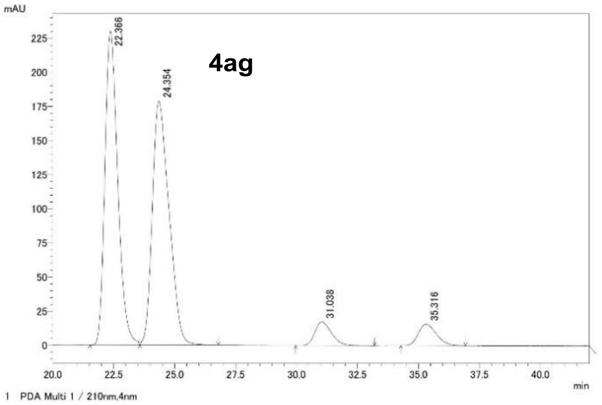

4ah

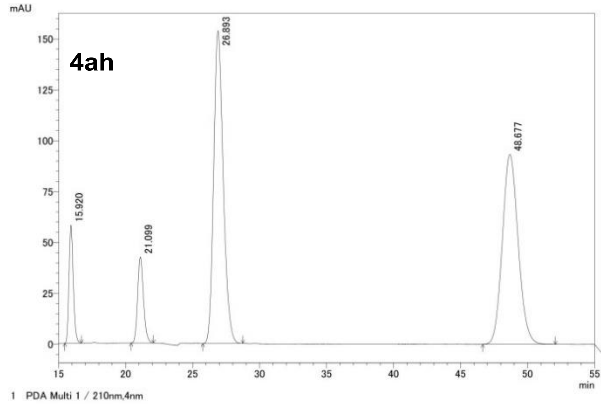

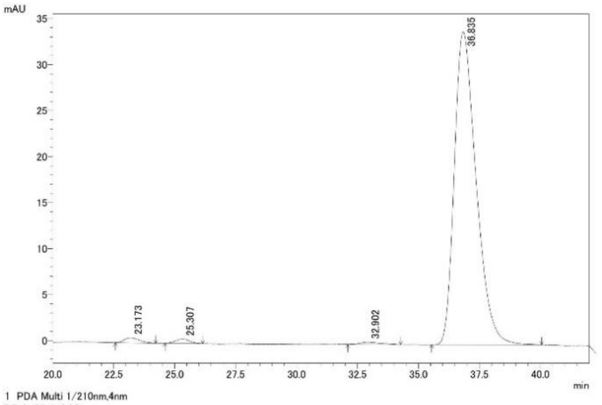

PDA Ch1 210nm

ピーク#	保持時間	面積%
1	23.173	1.117
2	25.307	0.906
3	32.902	0.626
4	36.835	97.351
合計		100.000

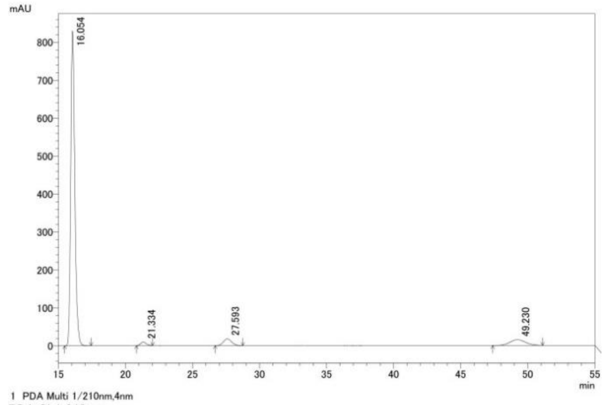

PDA Ch1 210nm

ピーク#	保持時間	面積%
1	16.054	88.523
2	21.334	1.389
3	27.593	3.934
4	49.230	6.153
合計		100.000

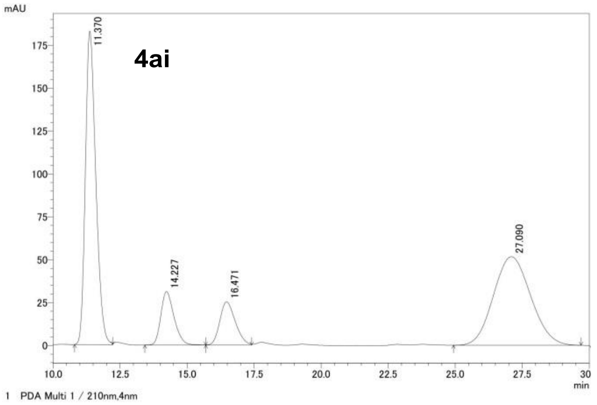

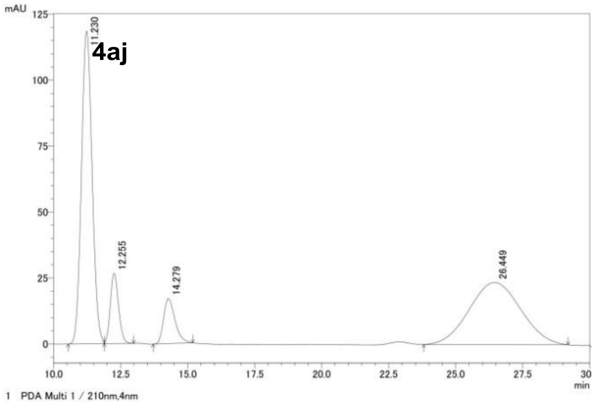

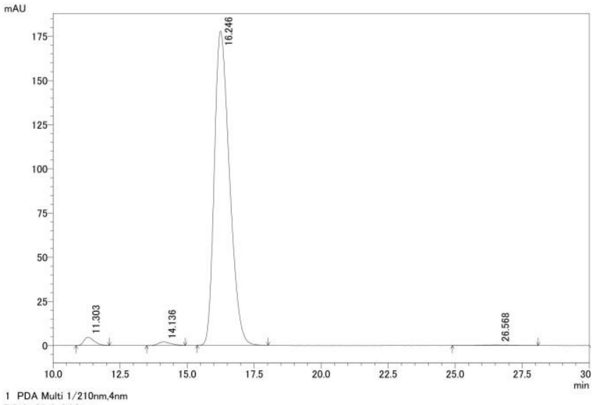

PDA Ch1 210nm

ピーク#	保持時間	面積%
1	11.303	1.709
2	14.136	0.907
3	16.246	97.135
4	26.568	0.249
合計		100.000

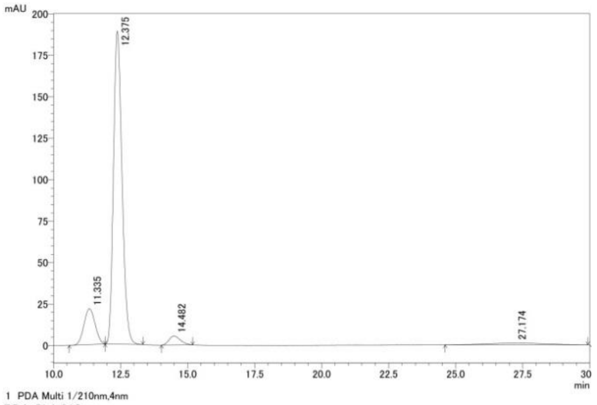

PDA Ch1 210nm

ピーク#	保持時間	面積%
1	11.335	12.143
2	12.375	81.692
3	14.482	3.102
4	27.174	3.063
合計		100.000

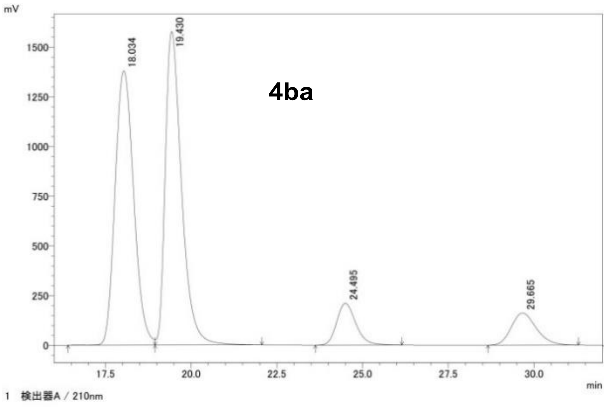

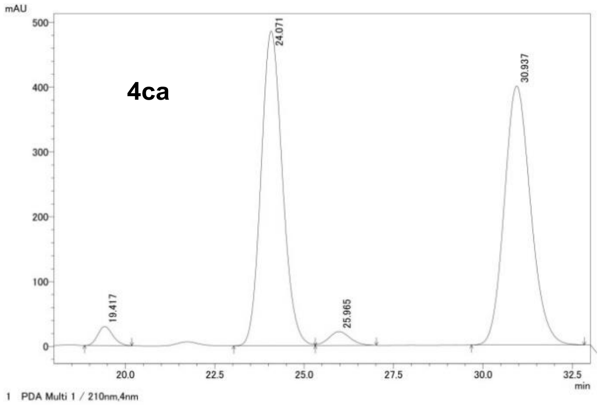

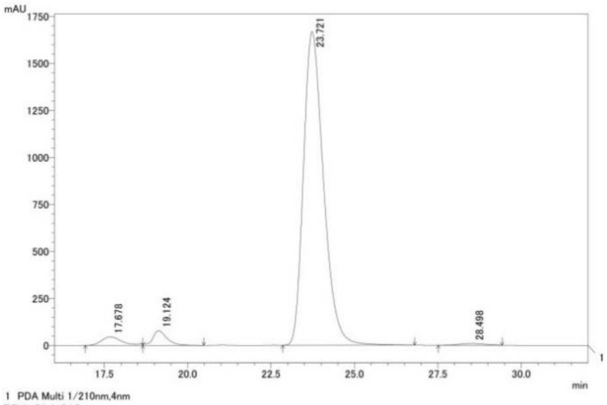

PDA Ch1 210nm

ピーク#	保持時間	面積%
1	17.678	2.589
2	19.124	3.083
3	23.721	93.711
4	28.498	0.617
合計		100.000

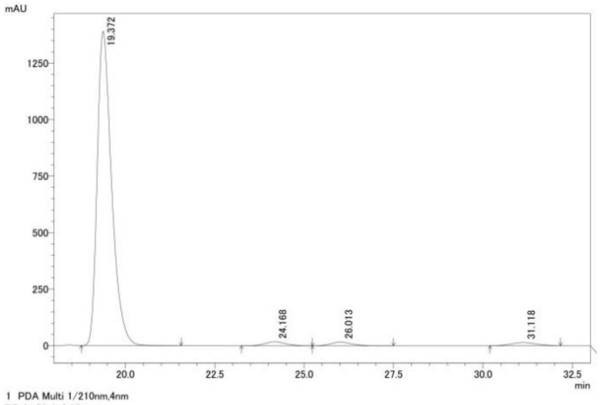

PDA Ch1 210nm

ピーク#	保持時間	面積%
1	19.372	95.106
2	24.168	1.666
3	26.013	1.561
4	31.118	1.667
合計		100.000

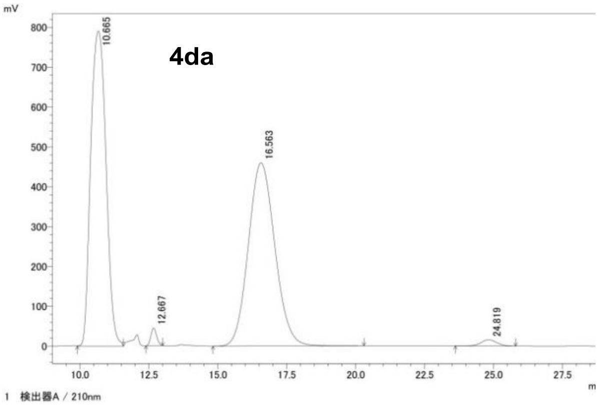

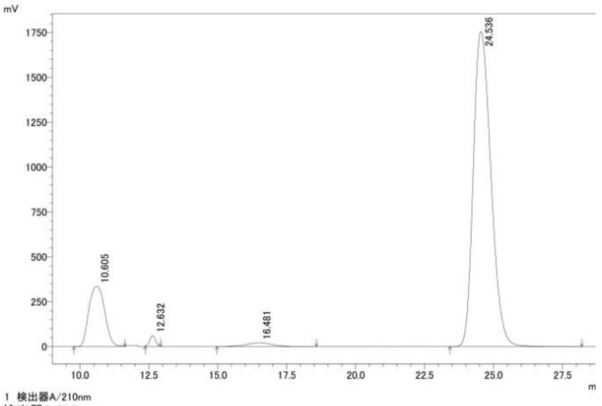

検出器A 210nm

ピーク#	保持時間	面積%
1	10.605	14.372
2	12.632	0.901
3	16.481	1.512
4	24.536	83.216
合計		100.000

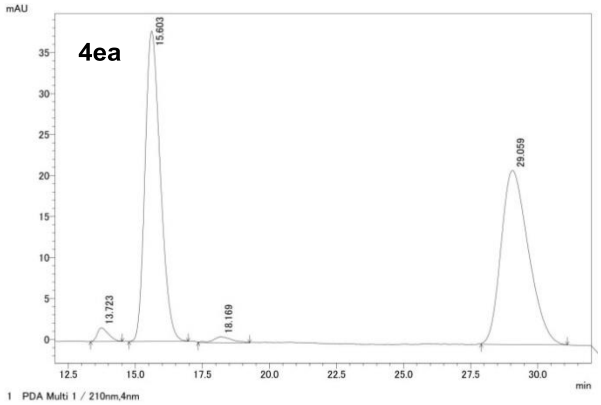

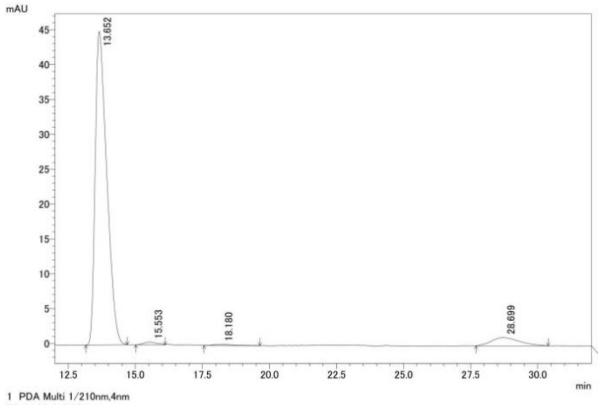

PDA Ch1 210nm

ピーク#	保持時間	面積%
1	13.652	92.463
2	15.553	0.971
3	18.180	0.569
4	28.699	5.997
合計		100.000

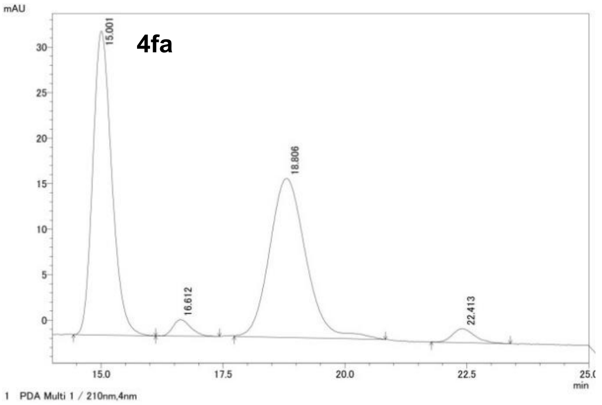

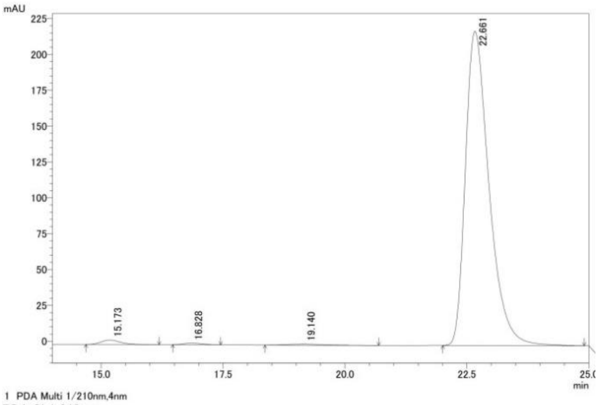

PDA Ch1 210nm

ピーク#	保持時間	面積%
1	15.173	1.156
2	16.828	0.379
3	19.140	0.534
4	22.661	97.931
合計		100.000

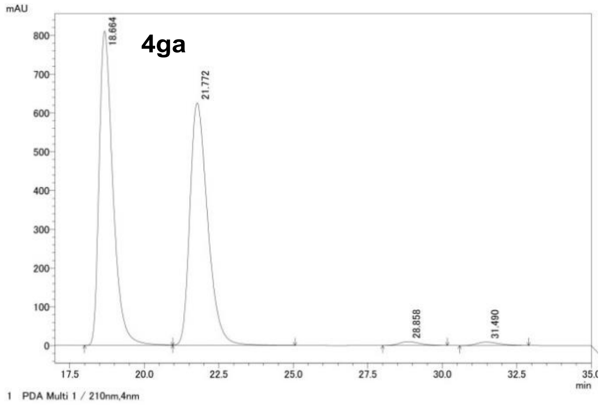

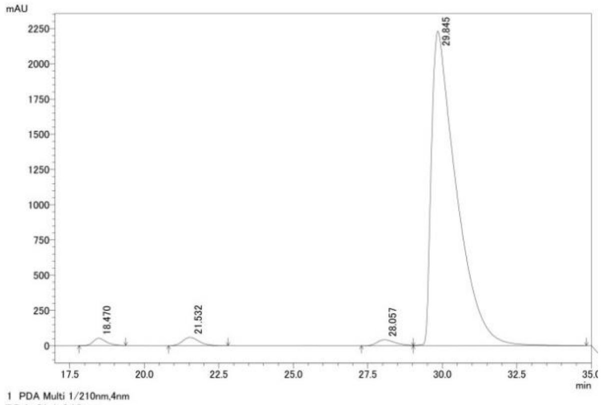

PDA Ch1 210nm

ピーク#	保持時間	面積%
1	18.470	1.148
2	21.532	1.660
3	28.057	1.320
4	29.845	95.872
合計		100.000

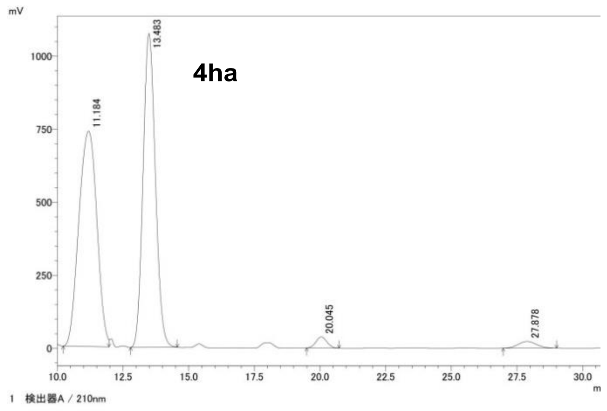

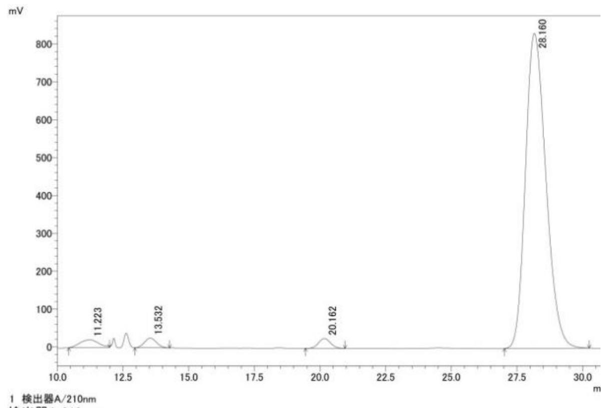

検出器A 210nm

ピーク#	保持時間	面積%
1	11.223	2.153
2	13.532	1.726
3	20.162	1.709
4	28.160	94.412
合計		100.000
